# Molecular Cloning and Expression Profiling of *CncC* in *Bactrocera dorsalis* Hendel

**DOI:** 10.3390/insects13090785

**Published:** 2022-08-30

**Authors:** Qianyan Fu, Tian Zeng, Yijuan Xu

**Affiliations:** Guangdong Laboratory for Lingnan Modern Agriculture, Department of Entomology, South China Agricultural University, Guangzhou 510642, China

**Keywords:** *Bactrocera dorsalis*, *BdCncC*, gene cloning, expression profiling

## Abstract

**Simple Summary:**

We cloned the *CncC* gene of *Bactrocera dorsalis* and performed a domain analysis of the protein to clarify the expression levels of the gene in different developmental stages and tissue sites of *B. dorsalis* and to provide a theoretical basis for further investigating the function of *CncC* in regulating pesticide resistance in *B. dorsalis*.

**Abstract:**

The cap ‘n’ collar isoform C (*CncC*) transcription factor is thought to be a regulator associated with antioxidant and detoxification genes that can enhance pest resistance by regulating the expression of detoxification enzyme genes. However, this transcription factor has not been well studied in the important agricultural pest *Bactrocera dorsalis*. In this study, the cDNA sequence of *CncC* in *B. dorsalis* was cloned, and the complete ORF sequence was obtained; it had a sequence length of 3378 bp, encoding a total of 1125 amino acids. Phylogenetic tree analysis showed that *B. dorsalis CncC* belonged to the CNC family and that its amino acid sequence showed the closest relationship with *B. tryoni*. The conserved structural region of *BdCncC* was analyzed and was found to include a conserved bZIP superfamily structural domain. Spatiotemporal expression analysis revealed that *BdCncC* was most highly expressed in the adult Malpighian tubules, followed by the antennae, foregut, and midgut, and then the brain, hemolymph, hindgut, and fat body. *BdCncC* was expressed at every developmental stage, and the highest expression was found in mature males. This study provides a theoretical basis for an in-depth investigation of the function of *BdCncC* in regulating pesticide resistance in *B. dorsalis*.

## 1. Introduction

Transcription factors (TFs) are members of the family of regulatory proteins. The sequences of TFs first contain DNA structural domains that recognize the corresponding cis-acting elements in the promoter regions of bound genes, followed by transcriptional regulatory structural domains, nuclear localization signal sequences, and oligomerization sites [1]. Due to the highly conserved structural domains of TF DNA sequences, they are classified into families such as bZIP, NAC, MYB, MYC, and WARK TFs [2,3]. The cap ‘n’ collar isoform C (*CncC*) TF is a member of the bZIP TF family and was first identified in Drosophila. It was then found to be directly homologous to the vertebrate protein nuclear factor erythroid 2-like Nfe2l2 (*Nrf2*) and the nematode protein skinhead family member 1 (*SKN-1*), which plays a key role in regulating cellular defense against oxidative stressors or electrophilic xenobiotics [4,5,6]. When oxidative stress is generated by exogenous stimuli, the conformation of kelch-like ECH-associated protein 1 (keap1) changes, releasing *CncC* into the nucleus and promoting the functional expression of *CncC* [7]. *CncC* has seven functional structural domains, where the main domain is Neh1, which contains a leucine bZIP structural domain for the recognition and binding of antioxidant response elements (AREs) [8]. Pests regulate the expression of downstream detoxification enzyme genes through the *CncC*/*keap1*-ARE signaling pathway, thereby increasing their resistance to pesticides. A study on the potato beetle (*Leptinotarsa decemlineata*) showed that *CncC* was required for the expression of 79% of P450 genes induced by imidacloprid, including genes encoding detoxification enzymes (P450, glutathione S-transferases) [9]. In *Tetranychus cinnabarinus*, *CncC* was found to affect the sensitivity of cinnabar mites to benzothiurin by regulating the expression of P450 genes [10]. Misra (2011) used resistant Drosophila species with significantly upregulated expression levels of *CncC* and P450 detoxification enzyme family genes after pesticide treatment. All genes related to detoxification showed a significant decrease after the RNAi knockdown of *CncC*, suggesting that *CncC* induces the expression of detoxification enzyme genes in Drosophila, thereby regulating pesticide resistance in Drosophila [6].

The oriental fruit fly, *Bactrocera dorsalis* (Hendel) (Diptera: Tephritidae), is a polyphagous pest that is considered highly invasive in horticulture. The oriental fruit fly infests hundreds of fruits and vegetables, including apricot, avocado, banana, citrus, coffee, etc. For the past 20 years, this fly has been introduced to and spread throughout sub-Saharan Africa [11,12]. Its existence has caused significant financial losses in orchard crops [13]. Furthermore, the overuse of insecticides to control *B. dorsalis* has led to the development of insecticide resistance [13]. Thus, the oriental fruit fly is an important quarantine pest. Chemical-based control strategies are still the main management method for oriental fruit flies, and resistance to these chemicals has become increasingly prominent in recent years, representing a major bottleneck in the sustainable control of infestation [14]. In this study, we obtained the ORF sequence of the *B. dorsalis BdCncC* gene by cloning, translated it into the corresponding amino acid sequence, and performed domain analysis to clarify its expression levels in different developmental stages and tissue sites of *B. dorsalis*, with the aim of providing a theoretical basis for an in-depth investigation of the function of *BdCncC* in the resistance of *B. dorsalis*.

## 2. Materials and Methods

### 2.1. Insects

*B. dorsalis* was reared at 27 °C with 75% relative humidity under a 14:10 h light:dark photoperiod. Adults were fed an artificial diet consisting of yeast extract and dry sugar mixed at a 1:1 ratio (*w*/*w*) and housed in transparent plastic cages.

### 2.2. RNA Isolation, Reverse Transcription, and BdCncC Cloning

Total RNA was isolated from adults using TRIzol^®^ reagent (Invitrogen, Carlsbad, CA, USA). RNA quality was checked by 1% agarose gel electrophoresis. Reverse transcription was performed using the PrimeScript™ II 1st strand cDNA Synthesis Kit (Takara, Dalian, China). After reverse transcription, the synthesized cDNAs were stored at −20 °C for future use. The reference sequence of the *BdCncC* cDNA was acquired from NCBI (GenBank number, KJ957012). Primers were designed (Table 1) using Primer Premier 5.0 (Premier Biosoft International, Palo Alto, CA, USA). PCR was performed using Q5 High-Fidelity DNA Polymerase (New England Biolabs, Ipswich, MA, USA) according to the manufacturer’s instructions. The purified PCR product was cloned into the pEASY-Blunt Zero Cloning Vector (TransGen, Beijing, China) following the manufacturer’s instructions before being sequenced. Positive clones were sent for sequencing by Sangon Biotech (Shanghai, China).

### 2.3. Analysis of the Protein Sequence and Biological Information of BdCncC

The obtained full length of *CncC* was compared with the Blast (https://blast.ncbi.nlm.nih.gov/Blast.cgi, accessed on 6 June 2022) in NCBI. The cloned *BdCncC* sequences were analyzed using DNAMAN 9.0. ORF prediction was performed using NCBI ORF Finder (http://www.ncbi.nlm.nih.gov/orffinder, accessed on 7 June 2022). The analysis of conserved structural regions was performed using NCBI Conserved Domains (https://www.ncbi.nlm.nih.gov/cdd, accessed on 7 June 2022). The analysis of protein functional structural domains was performed using SMART (http://smart.embl-heidelberg.de/, accessed on 7 June 2022). The molecular weight, isoelectric point, instability index (II), and aliphatic index were predicted with ProtParam (https://web.expasy.org/protparam/, accessed on 10 June 2022). Hydrophilicity prediction was conducted using ProtScale (https://web.expasy.org/protscale/, accessed on 10 June 2022). Signal peptide prediction was carried out using SignalP (http://www.cbs.dtu.dk/services/SignalP/, accessed on 10 June 2022). Transmembrane prediction was performed using TMHMM (http://www.cbs.dtu.dk/services/TMHMM-2.0/, accessed on 10 June 2022). Subcellular localization analysis was performed using PSORT II (https://www.genscript.com/psort.html, accessed on 11 June 2022). The prediction of 3D structures was conducted using SWISS-MODEL (https://swissmodel.expasy.org, accessed on 11 June 2022).

### 2.4. Phylogenetic Analysis and Identification

The *CncC* and *Nrf2* protein sequences were obtained from the NCBI web server (https://www.ncbi.nlm.nih.gov/, accessed on 20 June 2022) and aligned with the sequences generated in the present study using the Clustal website (https://www.ebi.ac.uk/Tools/msa/clustalo/, accessed on 20 June 2022). To infer evolutionary relationships, the neighbor-joining method was used to construct a phylogenetic tree in MEGA11.0.1 software with 1000 bootstrap replicates.

### 2.5. Reverse-Transcription Quantitative PCR Analysis

RNA was extracted from different tissues, including the brain, antennae, hemolymph, foregut, midgut, hindgut, Malpighian tubules, and fat body. TRIzol^®^ reagent (Invitrogen, Carlsbad, CA, USA) was used for RNA isolation. The extracted RNA was purified using the phenol/chloroform method and dissolved in RNase-free water. The purity of the extracted RNA was assessed spectrophotometrically by measuring the OD260/280 ratio, where an OD260/280 of 1.8–2.0 indicated good-quality RNA. RNA integrity was evaluated via electrophoresis on a formaldehyde agarose gel. The RNA (1 μg) was then reverse transcribed to cDNA using the PrimeScript™ RT reagent Kit with gDNA Eraser (Takara, Otsu, Japan) according to the manufacturer’s instructions. Biosynthesized cDNA was used as a template in RT–qPCR conducted on a C1000 Touch thermal cycler (Bio-Rad Laboratories, CA, USA) with TB Green Premix Ex Taq II (Tli RNase H Plus) (Takara Bio, Otsu, Japan). The thermal cycling conditions were as follows: 95 °C for 30 s, 40 cycles at 95 °C for 5 s, and 60 °C for 34 s. RT–qPCR was conducted with three technical and five biological replications. α-*Tubulin* and *RPL* were used as reference genes for gene expression analysis in *B. dorsalis* due to their expression stability.

### 2.6. Statistical Analysis

*CncC* gene transcript levels were quantified using the 2^−ΔΔCT^ method [15]. Experimental data were analyzed and plotted using SPSS 26.0 and GraphPad Prism 9.0. *BdCncC* expression pattern analysis was performed by one-way analysis of variance (ANOVA) with Duncan’s multiple range test. A probability value of *p* < 0.05 was considered statistically significant.

## 3. Results

### 3.1. Molecular Cloning and Sequencing of BdCncC

**Figure 1:**
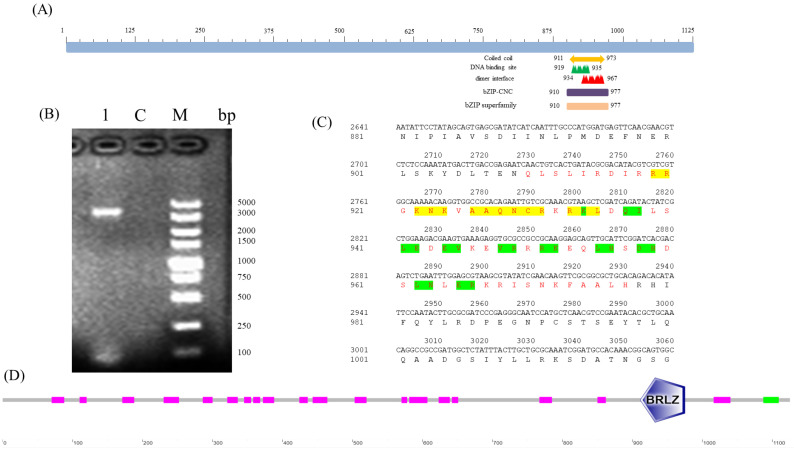
Functional domain prediction based on the *BdCncC* amino acid sequence. (**A**,**D**) Schematic illustration of *BdCncC*; (**B**) electrophoresis of *BdCncC* PCR products; M: DNA Marker DL5000; C: control group; 1: PCR product; (**C**) amino acid sequence structure of *BdCncC*, where red letters represent the conserved regions of the *BdCncC* protein, including the basic region domain and leucine zipper—yellow highlighting indicates the DNA-binding site, and green highlighting indicates the dimer interface site.

PCR amplification of *BdCncC* was carried out using the specific primers designed using the whole insect cDNA of *B. dorsalis* as the template, and electrophoresis showed that the amplified target fragment was consistent with the length of the target gene (Figure 1B). The results of NCBI Blast on the cloned sequence showed that it was the *CncC* gene of *B. dorsalis*. The analysis of the sequencing results using DNAMAN9.0 showed that the full-length *B. dorsalis BdCncC* gene was 3494 bp and encoded 1161 amino acids. The ORF of *BdCncC* was predicted to be 3378 bp long, encoding 1125 amino acids, according to NCBI ORF Finder, and the amino acid sequence was obtained by translation. The obtained ORF amino acid sequence was analyzed using NCBI Conserved Domains for the conserved structural region of *BdCncC*; the protein contains a conserved bZIP superfamily structural domain in which the conserved structural region is located between amino acids 910–977, with an E-value of 1.09 × 10^−32^. The conserved structural domain includes 14 DNA-binding sites and 19 dimer interfaces (Figure 1A,C). SMART was used to analyze the functional structural domains of the *BdCncC* protein, which includes two conserved functional domains: basic region and leucine zipper (BRLZ) domains (Figure 1D).

### 3.2. Analysis of Biological Information of BdCncC

**Figure 2:**
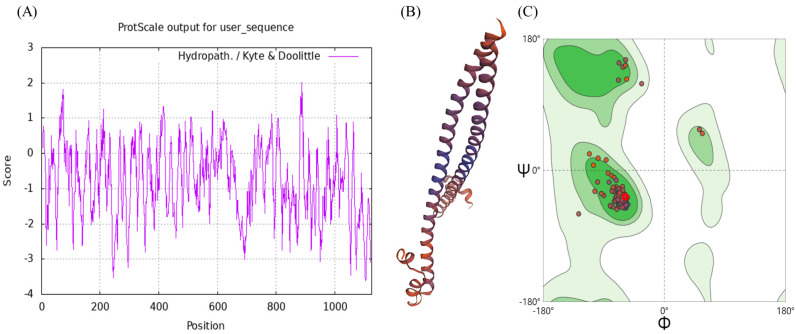

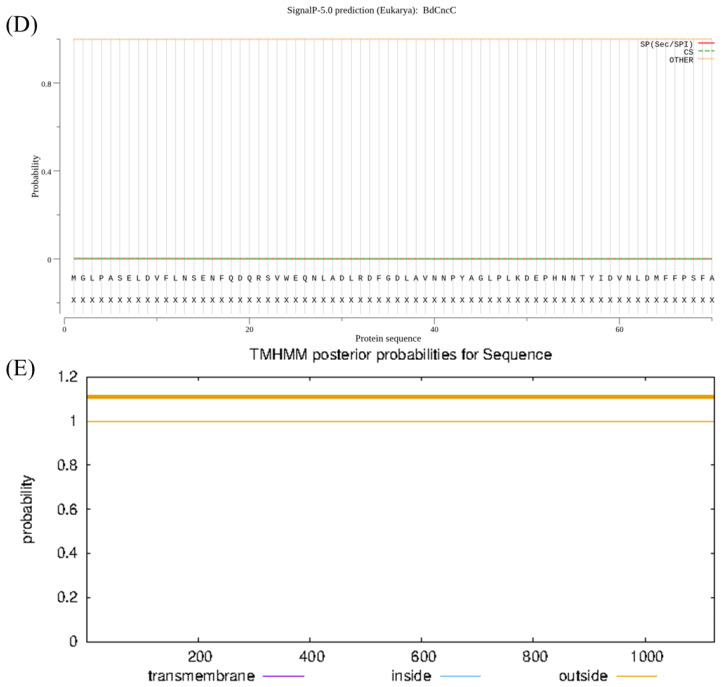
Domain analysis of *BdCncC*. (**A**) Hydrophobicity analysis; (**B**) three−dimensional structural model; (**C**) Ramachandran plots; (**D**) predicted signal peptide prediction results; (**E**) prediction of transmembrane domain structures.

The molecular weight of the *BdCncC* protein was predicted by ProtParam to be 121.42 kDa, indicating that it is a large protein; the isoelectric point (isoelectric point) was 5.17, indicating that it is an acidic protein; the instability index (II) was 47.11 (>40), indicating that it is unstable; and the aliphatic index was 61.15 (<90), indicating that it is a water-soluble protein. The highest Score value was 2.011 at amino acid position 886, and the lowest Score value was −3.611 at amino acids 1104 and 1105, whereas the grand average of hydropathicity (GRAVY) was −0.784, indicating that it is a hydrophilic protein (Figure 2A). PSORT II was used to predict the subcellular localization of *BdCncC* in nuclear, cytoskeletal, cytoplasmic, and mitochondrial fractions (Table 2). By applying PSORT II, the tertiary structure of *BdCncC* was predicted using TF MafB (PDB chain ID: 2wty.1A) as a template, and the Ramachandran favored value was 98.93%, indicating that the predicted 3D structure was plausible (Figure 2B,C). Signal peptide prediction using SignalP revealed no signal peptide in *BdCncC*, indicating that it cannot be secreted extracellularly (Figure 2D). Transmembrane prediction of the *BdCncC* protein using TMHMM predicted a transmembrane helix number of zero, indicating that it is not a transmembrane protein (Figure 2E).

### 3.3. Sequence and Phylogenetic Tree of BdCncC

**Figure 3:**
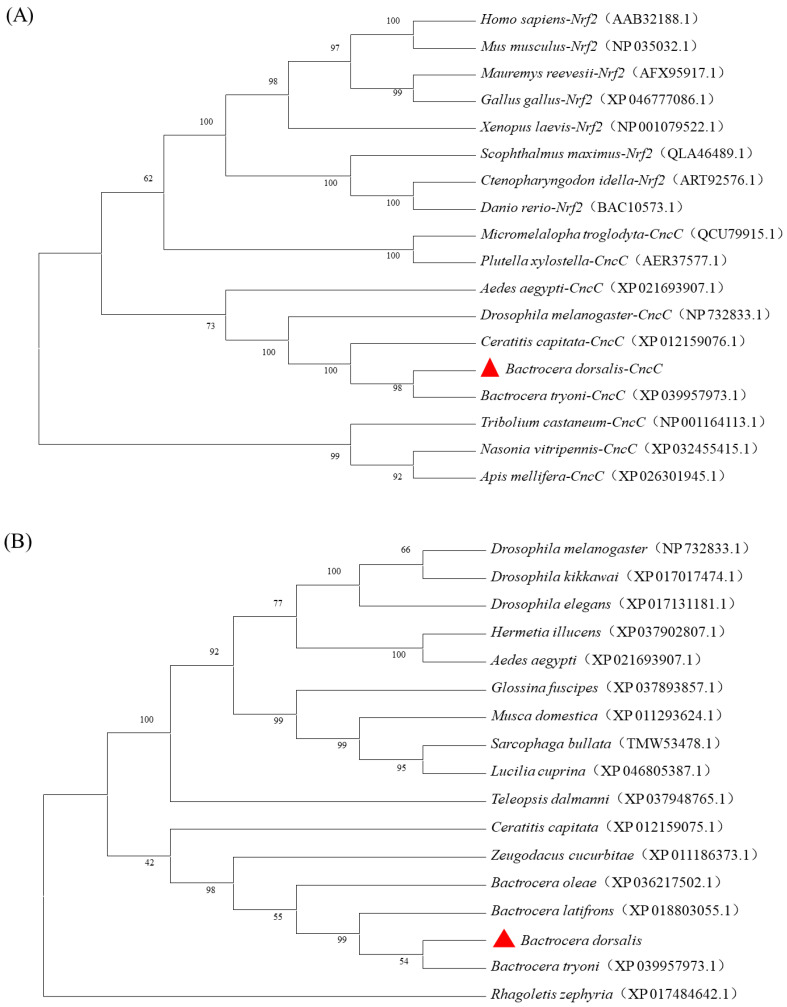
Phylogenetic tree of *BdCncC* generated with the neighbor-joining method. (**A**) Phylogenetic tree of *CncC* and *Nrf2*; (**B**) phylogenetic tree of dipteran *CncC* sequences. The numbers along the branches indicate bootstrap support from 1000 replicates; (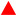) represent *CncC* in *B. dorsalis*.

The evolutionary tree constructed by comparing the sequences of insect *CncC* and Nrf2 from other species showed that *CncC* and Nrf2 were sister groups (Figure 3A). The phylogenetic tree of the *CncC* protein sequences of dipteran insects showed that *CncC* proteins were relatively conserved, and the amino acid sequence of *B. dorsalis BdCncC* presented the closest affinity to the amino acid sequence of *B. tryoni* cap ’n’ collar (Figure 3B). The evolutionary tree results suggest that *BdCncC* was a member of the CNC family. And the cladograms and the high similarity of the conserved regions in the multiple sequence alignment results proved the conservativeness of *CncC* (Figures S1 and S2).

### 3.4. Temporal and Spatial Expression Patterns of BdCncC

**Figure 4:**
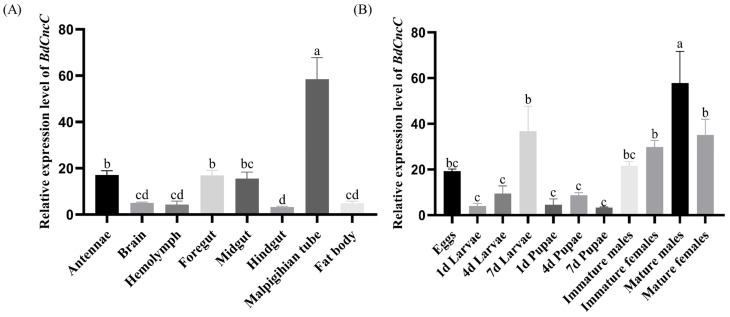
Expression profiles of the *BdCncC* gene in different tissues and developmental stages of *B. dorsalis*. (**A**) Expression profiles of the *BdCncC* gene in different tissues, and different colors indicate different tissue; (**B**) Expression profiles of the *BdCncC* gene in different developmental stages, and different colors indicate different developmental stages. Means and SEs from five biological replicates are shown. The different letters above the bars indicate significant differences in gene expression levels among treatments (*p* < 0.001, one-way ANOVA test).

The results of the analysis of *BdCncC* expression levels in different tissues and developmental stages of *B. dorsalis* using qRT–PCR showed that *BdCncC* expression was highest in the adult Malpighian tubule, followed by the antennae, foregut, and midgut, and then the brain, hemolymph, hindgut, and fat body (Figure 4A, F(7, 32) = 25.016, *p* < 0.001). Among different developmental stages, *BdCncC* was highly expressed mainly in adults, 7-day larvae, and eggs, and the highest expression was found in mature males. The expression levels were relatively low in 1-day and 4-day larvae and pupae (Figure 4B, F(10, 42) = 9.292, *p* < 0.001).

## 4. Discussion

The *CncC* TF protein family was originally studied in vertebrates, invertebrates, and postnatal animals. This TF is thought to be a regulator associated with antioxidant and detoxification genes [5]. In this study, the cDNA sequence of the *B. dorsalis BdCncC* TF was cloned, and the complete ORF sequence was obtained, showing a sequence length of 3378 bp and encoding a total of 1125 amino acids. The predicted protein molecular weight was 121.42 kDa. The conserved structural region of *BdCncC* was also analyzed, and it was found to include a conserved bZIP superfamily structural domain. Phylogenetic tree analysis showed that the amino acid sequences of *B. dorsalis BdCncC* were very similar to those of *B. tryoni*, indicating that they are more evolutionarily homologous to each other than other sequences in this family. Spatiotemporal expression analysis showed that *BdCncC* was expressed at every developmental stage of *B. dorsalis* and that its expression was highest in adults. Since the field control of *B. dorsalis* is usually performed by spraying insecticides on adults; the high expression of *BdCncC* in adults may contribute to the metabolism of insecticides or phytochemicals in *B. dorsalis* and, thus, enhance resistance. The tissue distribution of a gene is usually related to its function. *BdCncC* expression was highest in the adult Malpighian tubule. The Malpighian tubule is a multifunctional organ capable of playing roles in compound excretion, insect metabolism, and accumulated metal of harmful compound detoxification [16]. Higher detoxification enzyme activity is also associated with the malleolus [17,18]. Therefore, the overexpression of *BdCncC* in the detoxification organ of *B. dorsalis* may play an important role in insecticide detoxification, and further studies can be conducted to reveal the function of *CncC* in different developmental stages and tissues of insects.

In summary, we identified and analyzed the structural features of *BdCncC* in B. dorsalis. Numerous studies have shown that insects increase their resistance to drugs by regulating the expression of detoxification enzyme genes [19,20,21]. A study showed a significant increase in transcript levels of *BmCncC* and enhanced activity of GST and *CYP450* after 24 h of octreotide treatment. [22]. In Drosophila, the expression of the *GSTd7*, *GSTd2*, *CYP6A2*, and *CYP6A8* genes is significantly downregulated, and sensitivity to malathion is increased after silencing *CncC* [6]. The RNAi knockdown of *CncC* decreases *CYP6DA2* gene expression and increases the sensitivity of cotton aphids to cotton cotyledon phenol [23]. In *Spodoptera litura*, reactive oxygen species (ROS) activate the *CncC* TF to regulate the expression of the cytochrome P450 gene *CYP6AB12*, which mediates resistance to λ-cyhalothrin [24]. Therefore, it is hypothesized that *CncC* may mediate resistance in *B. dorsalis* by regulating the expression of multiple detoxification genes. Studying the functions of detoxification genes and the binding sites of TFs is important for understanding how *BdCncC* regulates the expression of resistance genes in *B. dorsalis*, and further studies on this topic are needed.

## Figures and Tables

**Table 1:** Primers used for *BdCncC* in the study.

Experiment	Primer Names	Sequences (5′-3′)
Full-length	*CncC*-F	ATGGGTTTGCCCGCTTCGGA
	*CncC*-R	TCAATCTTTCTGATGCGTTT
RT–qPCR	*CncC*-q-F	CGGTGCGAATAGTGCTTT
	*CncC*-q-R	TATGGATGTGTGGGATAGTGAG
Reference genes-1	*α-Tubulin*-F	CGCATTCATGGTTGATAACG
	*α-Tubulin*-R	GGGCACCAAGTTAGTCTGGA
Reference genes-2	*RPL*-F	CCCGTCATATGCTGCCAACT
	*RPL*-R	GCGCGCTCAACAATTTCCTT

**Table 2:** Subcellular localization prediction of *BdCncC*.

Predicted Location	*BdCncC*
Nuclear	87%
Cytoskeletal	4.3%
Cytoplasmic	4.3%
Mitochondrial	4.3%

## Data Availability

The data presented in this study are available upon request from the corresponding author.

## Supplementary Materials

The following supporting information can be downloaded at: https://www.mdpi.com/article/10.3390/insects13090785/s1, Figure S1: Multiple sequence alignment of conserved regions of insect *CncC* and *Nrf2* from other species; Table S1: Sequences of insect *CncC* and *Nrf2* from other species for the phylogenetic analysis; Figure S2: Multiple sequence alignment of conserved regions of *CncC* of diptera; Table S2: Sequences of diptera *CncC* for the phylogenetic analysis.
