# Supplementary material for: Molecular Cloning and Expression Profiling of CncC in Bactrocera dorsalis Hendel
Source: Insects. 2022 Aug 30;13(9):785. doi: 10.3390/insects13090785 (PMC9503647; doi:10.3390/insects13090785)
Supplement: Supplementary file 1 [file insects-13-00785-s001.zip › Table S1 Sequences of insect CncC and Nrf2 from other species for the phylogenetic analysis.pdf]

Table S1 Sequences of insect *CncC* and *Nrf2* from other species for the phylogenetic analysis.

>*Homo sapiens-Nrf2*（AAB32188.1）
MDLIDILWRQDIDLGVSREVFDFSQRRKEYELEKQKKLEKERQEQLQKEQEK
AFFTQLQLDEETGEFLPIQPAQHTQSETSGSANYSQVAHIPKSDALYFDDCMQ
LLAQTFPFVDDNEVSSATFQSLVPDIPGHIESPVFIATNQAQSPETSVAQVAPV
DLDGMQQDIEQVWEELLSIPELQCLNIENDKLVETTMVPSPEAKLTEVDNYHF
YSSIPSMEKEVGNCSPHFLNAFEDSFSSILSTEDPNQLTVNSLNSDATVNTDFG
DEFYSAFIAEPSISNSMPSPATLSHSLSELLNGPIDVSDLSLCKAFNQNHPESTA
EFNDSDSGISLNTSPSVASPEHSVESSSYGDTLLGLSDSEVEELDSAPGSVKQN
GPKTPVHSSGDMVQPLSPSQGQSTHVHDAQCENTPEKELPVSPGHRKTPFTK
DKHSSRLEAHLTRDELRAKALHIPFPVEKIINLPVVDFNEMMSKEQFNEAQLA
LIRDIRRRGKNKVAAQNCRKRKLENIVELEQDLDHLKDEKEKLLKEKGENDK
SLHLLKKQLSTLYLEVFSMLRDEDGKPYSPSEYSLQQTRDGNVFLVPKSKKP
DVKKN

>*Scophthalmus maximus-Nrf2*（QLA46489.1）
METEVMHPSQQDMDLIDILWKQDIDLGAQREVFDYNHRQKEHELQRQRKLE
EEKRLHLLREQEKALLAQLQLDEETGEYIPRPPPSSAPLQSTVTPLEVTQNVSF
TEENGDALSFDECLQLLAETFPVEETENTSVCLDTAAVSVASSGNMMMPPER
PPLPPAAISPGPLPPTLPPQRMSPDLEQAXMELLSLPELQQCLSMQMEDTLETT
TYSLPNNPKVQNANFTVYPMTNLKEGETNSLNVCPAELINTFDGSVSSMASP
DNLSQMEVKAPQLNTNFGVESFCDMFYPDTILEESSGQHGLEGNLSTTMSDV
TNKPSFTPMDLYSLSPGEAFDRGKQNLTAEVPDSDSGISSYTSPNACSPAKSA
YGDGSYGYSDSDMDEMDQSPGSAESDYSEMFSLNYQPDDLQTTDSASAIGQP
HQQHVKKPKHYKTDLAEESGHNNAPFTKDKLKRRSDVRLPRDEQRAKALKI
PLSVDMIINLPVDDFNEVMSKHQLTEAQLALVRDIRRRGKNKVAAQNCRKR
KMZNIVGLEGELDSLKDEKERLLSEKSQNITNLKEMKQQLNSLYLEVFSRLRD
EKGNSFSPSDYSLQQSTEGSIFLVPRIKKTLIKREENHLSPM

>*Ctenopharyngodon idella-Nrf2*（ART92576.1）
MMEIELPKMHPSQQDMELIDILWRQDVDLGAGREVFDFSYRQKEVELRRQR
EQEEEKRQQHLREQEKALLAQLQLDEETGEFVPRSLPASGTLTQTNTTNGEIA
QNGAFAAQEGDALSFDECMQLLAETFPLDEPAESAPPCLDVSVPPTTDLMMP
ADTPAFTQNPLLPGSLDQAWMELLSLPELQQCLNMQMQETLNMDGFMKPSG
EAQNPSYSQYLPGMDHLSSVQTEVCPPEYINTYDGSFNNMVSPNLSQMSLNV
PDVGAEFRPEEFNELFYPEMEAKVNSGPLTSDGGNMVSQLAETASDSPVNPM
DLQSFSPGNLSSGKPEPIVEFPDSDSGLSLDSSPHMSSPGKSLNEDGSFGFSDSD
SEEMDGSPGGTESDYTEIFPLVYLNDGAQTSLSDKSPAEQQEMKAKNPKTEP
VEASGYSKPPFTKDKQKKRSEARLSRDEQRAKALQIPFTVDMIINLPVDDFNE
MMSKHQLNEAQLALVRDIRRRGKNKVAAQNCRKRKLENIVGLEYELDSLRE
EKERLKKEKSERSTSLREMKQQLSTLYQEVFGMLRDEHGKPFSPNEYSLQHT
ADGTVFLVPRLKKTLVKNN

>*Mus musculus-Nrf2*（NP_035032.1）
MMDLELPPPGLQSQQDMDLIDILWRQDIDLGVSREVFDFSQRQKDYELEKQK
KLEKERQEQLQKEQEKAFFAQFQLDEETGEFLPIQPAQHIQTDTSGSASYSQV
AHIPKQDALYFEDCMQLLAETFPFVDDHESLALDIPSHAESSVFTAPHQAQSL
NSSLEAAMTDLSSIEQDMEQVWQELFSIPELQCLNTENKQLADTTAVPSPEAT
LTEMDSNYHFYSSISSLEKEVGNCGPHFLHGFEDSFSSILSTDDASQLTSLDSN
PTLNTDFGDEFYSAFIAEPSDGGSMPSSAAISQSLSELLDGTIEGCDLSLCKAFN
PKHAEGTMEFNDSDSGISLNTSPSRASPEHSVESSIYGDPPPGFSDSEMEELDS
APGSVKQNGPKAQPAHSPGDTVQPLSPAQGHSAPMRESQCENTTKKEVPVSP
GHQKAPFTKDKHSSRLEAHLTRDELRAKALHIPFPVEKIINLPVDDFNEMMSK
EQFNEAQLALIRDIRRRGKNKVAAQNCRKRKLENIVELEQDLGHLKDEREKL
LREKGENDRNLHLLKRRLSTLYLEVFSMLRDEDGKPYSPSEYSLQQTRDGNV
FLVPKSKKPDTKKN

>*Danio rerio-Nrf2*（BAC10573.1）
MMEIEMSKMQPSQQDMDLIDILWRQDVDLGAGREVFDFSYRQKEVELRRRR
EQEEQELQERLQEQEKTLLAQLQLDEETGEFLPRSTPLTHTPEADGGGAGEIT
QNGAFAEQEADPMSFDECMQLLAETFPLTEPAESAPPCLNTSAPPSTDLMMP
ADVPAFTQNPLLPGSLDQAWMELLSLPELQQCLNMPMQETLDMNAFMKPST
EAPTQNYSQYLPGMDHLGSAQTEVCPPEFTNTYNRSFNTMVSPNMNQLSLN
VPDVGAEFGPEEFNELFYPEMEVKVNNPPITSDGGNMVGDPPVNPIDLQSFSP
GDFSSGKPDPIVEFQDSDSGLSLDASPHMSSPGKSITEDGSFGFSDSDSEEMEG
SPGSMESDYNEIFPLVYLNDGSQTPLSEKSSTEKQEMKLKNPKMEPAEASGHS
KPPFTKDKLKKRSEARLSRDEQRAKALQIPFTVDMIINLPVDDFNEMMSKHQ
LNEAQLALVRDIRRRGKNKVAAQNCRKRKLENIVGLEYELDSLKEEKERLM
KEKSERSSNLKEMKQQLSTLYQEVFGMLRDENGKAFSPNEFSLQHTADGTVF
LVPRLKKTLVKNI

>*Mauremys reevesii-Nrf2*（AFX95917.1）
MELEAPPAPQDMNLIDILWRQDIDLGARREVFDFSQRQKEYELEKQKKLEKE
RQEQLQKEQEKALLAQLQLDEETGEFIPIQPAQHIESDNTGMPTNFSKTTHISK
PEADALSFDDCMQLLAETFSFVDDNEVFSAAFQVLVPAHIDSDPIFITSNQTQP
PESSVLQSPVTDVDNMENIEQVWEELLSIPELQCLNIENDNLAEVTTIANPETK
PSEIHSNFYNSLPITVNCNSDFLNTFEDSFSNILPPEDLSQLRVASLDTTSCLSSN
FSEDFFSTFVDPKVNGDTAAPNIVNKSFAEVPYEPIDISDFSRCKAFNGDLQGN
ALECNDSDSGISLNAHSNTASPEHSVESSVFGDAAFGYSDSEMEEMESAPGSL
HQSNAQMYSLQFHDPVPPSLGPSTQKSDLQCVNTPKRELPANPGHPKAPFTR
DKPSHYLEAHFTRDEQRAKALHIPFSVEKIINLPVDDFNEMMSKEQFNEAQLA
LIRDIRRRGKNKVAAQNCRKRKLENIVELEQDLGHLKDEKEKLLKEKGENDK
SIRLMKKQLTNLYLEVFSMLHDENGKPYSPSEYSLQQTKDGSIFLVPKSKKPE
TKF

>*Xenopus laevis-Nrf2*（NP_001079522.1）

MMEIEIPLPLQSQQDMDLIDILWKQDIDLGVSREVFDYNQRQKENDLEKQKK
LEKEKQEQLQKEQEKALYAQLQLDEETGEFIPMQQAAPTETVADTQALAGSI
QDKPSPVHELSFDECLKILAETFQIGPSNEDSPVAYQTLEPSAPIETNQIFFQSEP
NSVTAGTLNSIPAQGETVHEINQAWEELLSIPELQCLSNEIENMVEQSMYPNPE
STTMTETQNTFSFFTPLSAMEKPQENNTSVFPTDYVDTIASSVPSVNTNATFN
VESFCDDIFTLVDPKVTNIVPLTNNSGQLLKELLHDNVDITDLSLCKAFNGSN
QPEFNDSDSGVSVNTSPCATSPSQSLGGSAYGEPHYGYSDSDMEDMDSTPEIA
QENPPDNFTAPFTEDTYFSLSPFISHDTDPFEVKAHDSPAKEIPASPGYSKAPFA
KDKSLNLQEARFTRDEQRAKVLNLPFTVEKIVNLPVDSFNEIMSKYQFNEAQL
ALIRDIRRRGKNKVAAQNCRKRKMENIVELETDLDTLKYEKEKLLAERGEYN
NSLSQLKKNLGNLYMEVFNKLQDENGKPYSPQEYSLQQTKEGNIFLVPKTKK
VSIKKE

>*Gallus gallus-Nrf2*（XP_046777086.1）
MNLIDILWRQDIDLGARREVFDFSQRQKEYELEKQKKLEKERQEQLQKEREK
ALLAQLVLDEETGEFVPAQPAQRVQSENAEPPISFSQSTDTSKPEEALSFDDC
MQLLAEAFPFIDDNEASPAAFQSLVPDQIDSDPVFISANQTQPPSSPGIVPLTDA
ENMQNIEQVWEELLSLPELQCLNIENDNLAEVSTITSPETKPAEMHNSYDYYN
SLPIMRKDVNCGPDFLENIEGPFSSILQPDDSSQLNVNSLNNSLTLSSDFCEDFY
TNFICAKGDGDTGTTNTISQSLADILSEPIDLSDFPLWRAFNDDHSGTVPECND
SDSGISLNANSSIASPEHSVESSTCGDKTFGCSDSEMEDMDSSPGSVPQGNASV
YSSRFPDQVLPSVEPGTQTPSLQRMNAPKKDPPAGPGHPKAPFTKDKPSGRLE
AHLTRDEQRAKALQIPFPVEKIINLPVDDFNEMMSKEQFSEAQLALIRDIRRRG
KNKVAAQNCRKRKLENIVELEQDLSHLKDEREKLLKEKGENDKSLRQMKKQ
LTTLYIEVFSMLRDEDGKSYSPSEYSLQQTRDGNIFLVPKSRKAETKL

>*Bactrocera dorsalis-CncC*
MGLPASELDVFLNSENFQDQRSVWEQNLADLRDFGDLAVNNPYAGLPLKDE
PHNNTYIDVNLDMFFPSFASAVAGGVAVKEETVGAGALNDTFPEPADIASIKT
EQLDKEEEEFVVENEVATASSTSDEAAPKIKKETESGEDNGQAAASEEFDLSP
FISQLDLFKEPSFEGKDGDDAGDIDDEWAGIPFTVDNETGEYIRLPLDEILNDV
LQLAEFGNINNECSNDSVASTSKAAAAKTNNNNNNNDKTKTETGEDELNEN
KNVSAIISNGKDEDLAKKTLGTITDGTTKEEHDKTEEDYIDDFDLSLSEIEVNS
LPSPLFDLDDEAREELADFDIMLQSAGAPPSPFHHHHSHQRGAGMQGYVGGG
ANSAFHRQAGGFHHGHHQGRMSLSRGVSMEQRLQDIASFFNPISSMGVVGG
VSDMPPYPHYPTHPYSYQGAAGIPAPPQHAQYPPPPPPHHHSHHAMLHANAT
LGDICPTQPHYGHNLGSAVTSSMHLTNSSHEADAGAAASAGAAGGNVGAYK
MEHDMMYYTNSSSDMNHTTEGFINSILNDEDLHLMDMNDSFCRMVDVNNS
TSNNSSVLGGSAGLSNGHASAGGSSATGSSTNGLAMNGAGATNSALAGGMT
ADLLGSGAGGTSASGDRLDASSDSAVSSMGSERVPSLSDGEWGEGSDSAQD
YHQGKYGRPYDYSYNNNNNRMNDGTRQPPVAQKKHQMFGKRFLNEQPDL
GAVPTTADNGSNIGVVPPTGTNSLLNIGPAQSIKYEYEAYPTVPGLQPGGAGG
ALNAEGAIGPVLSNKEQQAYDLKYGPAVAGAMGGYTLDFAQRPPRTPHEIV
QHNHTYTLPQGTGSMPRPQARDKKLSTSSSKSSKSVNGEEEHLTRDEKRARA

LNIPIAVSDIINLPMDEFNERLSKYDLTENQLSLIRDIRRRGKNKVAAQNCRKR
KLDQILSLEDEVKEVRRRKEQLHSDHDSLNLERKRISNKFAALHRHIFQYLRD
PEGNPCSTSEYTLQQAADGSIYLLRKSDATNGSGNISNSSSTSSSSSGSGSVSTL
LSHNGMSPQQQQQQQQHAMLGTATHLQQHHHHHHNAQAPQQQQQLSHMH
TQQVQQAQQQQQQQQQQRQHSAHAHAHHHQTHQKD

>*Micromelalopha troglodyta-CncC* （**QCU79915.1**）
MTRGPDSPTLSILRQSLMRSMSVEQRWQDLASLLTIPPPPDQYQHYHHHHHQ
HPHNISAHGAAGGYGPNHHAPLPPPAPEKHPDAYGATAPIDGAYKVESARHS
QQHDGIYYQNPSGEMAPPPNQDGLLQSILNDEDLQLMDMAMNEGMYTMRM
LDSAASHHAPHPNHTHMMMTAERDSASDSAVSSMGSERVPSLSDGEWCDGS
DSAQEFHSSKFRPYDAAYPRERSAPHQPPQKKHHMFGKRCFQEQQPAPAIEP
VPPRPGVVKYEPEQTYHHEPMHNMHNRIPLEVQDIINLPMDEFNERLSKHVLS
EAQLSLIRDIRRRGKNKVAAQNCRKRKLDQITSLADEVRTVRDRKHRTQREH
SQLLAERQRVKERFASLYRHVFQNLRDPEGRPLSSAAYSLQQAADGNVVLVP
KMPQHHDHPMNRSSDDEMDRKAKHYDQ

>*Plutella xylostella-CncC*（**AER37577.1**）
MLLDEVMPTMRSLSPDPEDMANVSRKCNNTLDMDLIEVLWKQDVDMGFSL
EDPVQPEGPTATKVLGDEIEKELKLAEEKIVKKNEEKAEIEKVKASLLDPEIKE
DDPWAGLSYTVDTETGEYVIQGDLPRELVSSEESRFDLLEEALGLVELGDEA
DTKNEPPEAIEGSSSGTGSEMLHPAMRHVPHHPLAHYHNQSLMRSMSVEQR
WQDLASLLTIPPPPPPEQYQHYHPHHHQHHAHNISSHGAAAGYGPKLHASIPP
VPEKHPEAYGGRSAPLEGAYKVEAAQHPQQHDPLYYQNASAEMTGPTNQD
GFLQSILNDEDLQLMDMAMNEGMYTMRMLDGAGPHHAPHHTHSHMMTTE
RDSASDSAVSSMGSERVPSLSDGEWCDGSDSAQEFHSSKFRPYDSSFGRERSN
PHQPQKKHHMFGKRCFQEQPAPTLDPVGRPSVVKYECPEQTYHHENMHMH
NVEFAPRPQLAPPHVTNLHAPALDLNTAHSSHALLQSNIPSPAPPRFAYATPER
VRHNHTYSAPLANDQRPAAVRDKLVRRLTDGSMSDGGSSATSSGQHLSRDE
KRAKALGIPLEVQDIINLPMDEFNERLSKHDLSEAQLSLIRDIRRRGKNKVAA
QNCRKRKLDQITSLADEVRTVRDRKQRTQRDHSNLLAERQRVKERFAALYR
HVFQNLRDPEGRPLSSQQYSLQQAADGNVVLVPKMPQHQDHPMNRTSDDD
MDRKAKSYDQ

>*Drosophila melanogaster-CncC*（**NP_732833.1**）
MISNKKSYAMKMLQLALALSLLHYNPDYLLHRWDSQLELGTHGDGWELEM
LRTVHRLDMDHNPYGNRKGLSPRIEDLLNFDDPSLGGMANGIGGCKLPPRFN
GSTFVMNLHNTTGNSSVQTAALQDVQSTSAAATGGTMVVGTGGAPTSGGQT
SGSALGEIHIDTASLDPGNANHSPLHPTSELDTFLTPHALQDQRSIWEQNLADL
YDYNDLSLQTSPYANLPLKDGQPQPSNSSHLDLSLAALLHGFTGGSGAPLSTA
ALNDSTPHPRNLGSVTNNSAGRSDDGEESLYLGRLFGEDEDEDYEGELIGGV
ANACEVEGLTTDEPFGSNCFANEVEIGDDEEESEIAEVLYKQDVDLGFSLDQE
AIINASYASGNSAATNVKSKPEDETKSSDPSISESSGFKDTDVNAENEASAASV
DDIEKLKALEELQQDKDKNNENQLEDITNEWNGIPFTIDNETGEYIRLPLDELL

NDVLKLSEFPLQDDLSNDPVASTSQAAAAFNENQAQRIVSETGEDLLSGEGIS
SKQNRNEAKNKDNDPEKADGDSFSVSDFEELQNSVGSPLFDLDEDAKKELDE
MLQSAVPSYHHPHPHHGHPHAHPHSHHHASMHHAHAHHAAAAAAAHQRA
VQQANYGGGVGVGVGVGVGVGSGTGSAFQRQPAAGGFHHGHHQGRMPRL
NRSVSMERLQDFATYFSPIPSMVGGVSDMSPYPHHYPGYSYQASPSNGAPGT
PGQHGQYGSGANATLQPPPPPPPPHHAAMLHHPNAALGDICPTGQPHYGHNL
GSAVTSSMHLTNSSHEADGAAAAAAAYKVEHDLMYYGNTSSDINQTDGFIN
SIFTDEDLHLMDMNESFCRMVDNSTSNNSSVLGLPSSGHVSNGSGSSAQLGA
GNPHGNQANGASGGVGSMSGSAVGAGATGMTADLLASGGAGAQGGADRL
DASSDSAVSSMGSERVPSLSDGEWGEGSDSAQDYHQGKYGGPYDFSYNNNS
RLSTATRQPPVAQKKHQLYGKRDPHKQTPSALPPTAPPAAATAVQSQSIKYE
YDAGYASSGMASGGISEPGAMGPALSKDYHHHQPYGMGASGSAFSGDYTVR
PSPRTSQDLVQLNHTYSLPQGSGSLPRPQARDKKPLVATKTASKGASAGNSSS
VGGNSSNLEEEHLTRDEKRARSLNIPISVPDIINLPMDEFNERLSKYDLSENQL
SLIRDIRRRGKNKVAAQNCRKRKLDQILTLEDEVNAVVKRKTQLNQDRDHLE
SERKRISNKFAMLHRHVFQYLRDPEGNPCSPADYSLQQAADGSVYLLPREKS
EGNNTATAASNAVSSASGGSLNGHVPTQAPMHSHQSHGMQAQHVVGGMSQ
QQQQQSRLPPHLQQQHHLQSQQQQPGGQQQQQHRKE

>*Ceratitis capitata-CncC*（XP_012159076.1）
MISNKKCYAMKMLQIALALSLLHVDPEQRLRNWDSQLELRDGDGWELEML
RAVPMVEYPYANRKTMMPLIEDLIRYDRQHDPTPPQYNVTAYMLNVQNDG
GTNQTATLPEIQATSSGTTNPASNVAVAGATALEQAANAAAAAAAASTNAS
NMGLPASELDVFLNSENFQDQRSVWEQNLADLRDFGDLAINNPYAGLPLKD
EPYNNTYIDVNLDMFLPSFASAAAAAAAAANDVKQENVAAGALNDTFPEPA
DVASIKTEQIDKEEAEEQEFVLNNEASSSGVSSAETVFTTTPTSQAAIIKKEPIE
SSGDDEAQTEMEEVDLSPFFSQLDIMKEESEIIDVLWKQDVDLGYSLTPAHLI
NTTGNNGSAGAVSTDDEIEKLKALEALKLDKPNFDGKDGGDDAGVPDIDDE
WAGIPFTVDNETGEYIRLPLDEILNDVLQLAEFGNLNNEPNNDDSYASTSKAA
AVKNDDDKRITKSETSEDEGINDDNITSARLVNGKEETEKDSNKKTLATSTTD
KDTKLERESINENDYIDDFDLSLSEIEVNSLPSPLFDLDDEAREELADFDMMLQ
SAAAPPPTFHHHHPHSHQRGAGMQGYVGGGANSAFHRQASGFHHGHHQGR
MSLSRGVSMEQRLQDIASFFNPISGMGVVGGVSDMPPYPHYPTHPYSYQSAA
GIPAPPPQHAQYPPPPPPHPHHPHSHHAMLHANATLGDICTPTQPHYGHNLGS
AVTSSMHLTNASHDADGGASASAGAAGGNVGAYKMEHDMMYYTNASSDM
NHTTEGFINSILNDEDLQLMDMNDSFCRMVDVNNSTSNNSSVLGGGLAAGH
ASTGGGSATGSIANGLTMSGAGAANSALTGGMTTDLLSSGAGATSAGGDRL
DASSDSAVSSMGSERVPSLSDGEWGEGSDSAQDYHQGKYGRPYDYSYNNNS
NNRMNDGTRQPPVAQKKHQMFGKRFLNEQPGLGAVTARPDNNSNAGGGVN
VLPPTGANNLLNIGPAQSIKYEYEPYPTVPGLQQGGSGGVLNADGAMGPALS
SKEQQAYDLKYGPAVAGAIGGYSLDFAQRPPRTPHEVVQHNHTYTLPQGTGS
MPRPQARDKKLSISSSKSSKSMNGEEEHLTRDEKRARALNIPISVSDIINLPMD
EFNERLSKYDLTENQLSLIRDIRRRGKNKVAAQNCRKRKLDQILSLEDEVKAV
RSRKEQLYADRDSLTLERKRISNKFAALHRHIFQYLRDPEGNPCSTSEYSLQQ

AADGSIYLLRKSDATNTGGNASNSSSTSSSSSGSSSSAGGGGGGGAAAAAEG
VNGHTMRHNGVSPQQHAMLGAATHLQQQQQQQTLHHQHHHAQTQQQQLS
HMHPQQAQQQQQQQQQQQQRQHSAHAHAHHHQTQQKD

>*Aedes aegypti-CncC*（XP_021693907.1）
MAALEYEDLPRMQSLTPEPDDTGSLKDGMGAPEFEQQQQQATTQVVVIGGD
SSCNLADDCCNDKPNSCDHDEVGYHSPLYNYSKYNLKEMDLIEVLWKQDVD
LGFTLTPLTVPSGASGGATSGMATKGATSAEDSSDDLEKLKVLLEIKHDDDK
KKDNADKQAEENPWAGMSYTVDIETGEYVLNATDGSEATLPPPLADLFLEE
GLNLPELINDTVIEETEEPAKKKTTAEQEEEEESEVKRIERELESSADELCQGA
AASVSATSGSTSASPCDLNICSEQQATATSSVVDREQQDSPNHPEAAGAASPD
DLEDILCDMMIQTSSSHFQHPRQPMQNYGHGMHGSLRQGGYHHHHHHHHH
QSRVPLSRAVSMEQRFQDLANLLSFPPGMGVGMGVGEMPPAHPHPHYPPHY
SYQANGAIPQHGQYHSHAVLQNASLADIGPTQPYYAPNLGSAVATSMHLTNS
TSETDAGATGYKMDHEMMYYSNTSSEMNHTDGFLNSILDDDLQLMDIAVNE
GMYTMRMLDHNATSSNSSVLGGSLGGSAAAVAAAAAAGLNGPAHLGGLMS
SASAAVSSGAMQTSLNGSTGTHGATGGTTSGDRLDASSDSAVSSMGSERVPS
LSDGEWGDGGSDSAQEYHNKYGGPFDYSYSGSNRLGDGTRQPPVAQKKHH
MFAKRYFQEQNTSIPSLPSATNPSATGPTVDPQSQLNASIPIKYEFDYMNPASL
SHLEGAVGPVTKQEDQTSAHNNPLSSVDMKYPYSLDFSRQNPASAPAARSHH
HDVIHHNHTYTLPHNSGANPKPQTRDKRIRKAEEEHLTRDEKRARALQIPIPV
QDIINLPMDEFNERLSKYDLSETQLSLIRDIRRRGKNKVAAQNCRKRKLDQIV
TLADEVKDMKMRKERLLRDREIIQTERKRIRDKFSALYRHVFQNLRDSEGNP
YSQEHWSLQQSADGTVVLVPRSVDRQQDLTGSNHQQQQQQLGMAGGSGLP
HSHHQQQHLLHLQQHQHPHSQQQHHHGGSSSGSNSTNSSGASGGSQHRPKE

>*Tribolium castaneum-CncC*（NP_001164113.1）
MDMDLIEVLWKQDVDLGFSLDVANGKADKPDADLQNGSPSSSISMENDEIE
KLKTLKAINDDNIKEEPENELADPWAGFNYTIDTETGEYVVKAEELSESLTGA
DCGPSCDLPLGDLSLPLPEFLLDEALRLVELDDTPQEAINVKELENLAEGTNET
EPSTSAPKASETPSSSKDSDDDLSILTDMIQTSQFHHPHHRAFQGRMPFVRTVS
MEQRWQDLANLLSLPSPGDTSGMPHPFSHHHPLHNYSHPHAHGMAYSPEAT
RGVLLHNATLTPPMGDINATVPYNNIGGTNLGNAVATSMNLTNSSEPMGEPN
SAPHYKLEPSHDMMYYQNSTTELNQTDGFLSSFLNDEDLQLMDMAMNEGM
YTMRMLDSNNAVSNLSMNGATGTTTSLSRTDVERMDTSSDSAVSSMGSERV
PSLSDGEWCDGGSDSGHTAGDHYVTDYHQTKYRPYDYSYTSRQHASALAA
ASDATRMPPVAQKKHQMFGKRYLQEQGATSAVSHQPPVKYEYRDPTAAPY
NSSQPEGAVGPKPSEMKYSCSMEFGRHSHLVRNSLDHIQHNHTYHLPTESTG
AMQRPVSRDKTKSKKGDEEHLTRDEKRARALNVPITVDDIINLPMDEFNERL
SKYDLSEPQLSLIRDIRRRGKNKVAAQNCRKRKLDQILSLADEVKDMRDRKM
RLMNEHEYVTGECQRMKDKYQQLYRHVFQNLRDPDGNQYSPYQYSLQTSA
DGSILVVPRSNSTMTNPEHKEPPPQGHKD

>*Nasonia vitripennis-CncC*（XP_032455415.1）

MLERITAKNMLNLKKIYSDELLQLALLLSLLRVDPDSYLGLDMQTIGVGSLDL
NNGSGWHTDSHTIVHRPAFVHPKNLDSMLLNYERDLIEDLNSLGRYNRINTA
PNDIHAYLLNVDDRAGPSTPVITDATPSTRNASSPDAPESSQTTEDTLGAAELT
QEDMDLIEVLWKQDVDLGFTLVDQTPKTDPATSSKKESEDEIEKLKTLEAINA
SDDKKDPKIKEEPAKEEDPWAGLPYTVDLETGEYILDDSAQQAAPSAEEAAA
PREQPKEDEEEQLLGEAGLGLGSVDELVGLGENSLGLNGTLGLENDFTSDLL
GDDCLLDGCSAVDGFLNNDSLGLPDGFNLEEALQLVGLDEVELEKKPEVKK
KREEDSDGARSESRTSTSENDEDCCCCNGCCPEGAEADSMIHTPQYHHPHHP
HHRSFQSRVPFVRSVGMDQRWPDIAPFFSVSGAGNGAAAAAASTDHFAHPV
GHHHHHHHHHHHPSYTSHGPAGATHHGHYEAQRNVLLHNATLAPPVGDLN
STGTYHNAGGPSNLGSAVATSMNLTNSSEPMGADSSGAYKPEPSDMIYYQN
ASADTMNQTDIFNALLDDPLGWNEDLSFNVSDGLYGGASTATGAQATLPGV
QAANASSGAGGGVVPGAVTDERMDASSDSAVSSMGSERVPSISDGEWMETG
SNSSHTQADSHYSMDYNGKYRMPYDACGYTLPSGRGGQRSSCQEARGGLAP
VAQKKHHMFGKRCFQEQGPMSGAAGGAGSPLAGQTHQAATTPGKYDYEAA
QSVAAGPPGQAYAGPIEGAAGPQPEIKYSCSLDFTRHQTARSSLEHVQHNHT
YHLPPESSGALQRPVSRDKKGRKSDGEEHLTRDEKRARALNVPIAVNDIINLP
MDEFNERLSKYDLSEPQLSLIRDIRRRGKNKVAAQNCRKRKLDQINSLSDEVK
EMRNRKMRLLSDRNYMLQEVARVKEKFGQLYRHIFHSLRDPEGRPYNSLEY
SLQQSATDGSILLVPRSGNNQSNGHYTRSTMDPSKPNKADSHHQPRQDHE

>*Apis mellifera-CncC*（XP_026301945.1）
MDLIEVLWKQDVDLGFTLVEPTTTATKKLSTVEKGSDDEIEKLKALEAINGSN
EEKDTKEYDEAQDDPWAGLPYTIDLETGEYILNSGNQEGDGNNAIEEDDRLL
REASLDLDNHPLAGLTDDSLGLTDTLELENDLPSDLLGGSLLASANVESLLNN
DSLDLPDGFNLEEALQLVGLDEAQSEETKPEVKKKEKDSIEESTSEAKDDESPI
ISSSSSVEVAKSSRCEDPETGDMIHTPQFHHPHHPHHRSFQGRMPFMRAMSME
QRWQDLASLLSLPGAPEHFPHTHPGYPGHGISHSHYEAQRNVLLHNATLAPP
VGDLNSTSPYHNVGGSSNLGSAVATSMNLTNSSEPMGAESGAAYKSEPADM
MYYHTPTSDSINQTTDGFLSSLLNDEDLHLMDMAMNDGMYTMRMLDNGNN
NASGPTGAAALSGVQTAGGTTSSATGVTTLPGVTDERMDASSDSAVSSMGSE
RVPSLSDGEWMETGSNSSHTQADSHYTMDYASKYRMSYDCSYSVSGRNAGS
PRCQTERTMPPVAQKKHQMFAKRYFQEQGTGSPLGATAHPTTPMKYEYDSH
TVGAGAPGNAYSGPIEGATGPQPEIKYSCSVDFSRHQSGRSAIEHVHHNHTYH
LPAESSGSLQRPVSRDKKVRKNDGEEHLTRDEKRARALNVPIPVNDIINLPMD
EFNERLSKYDLSEAQLSLIRDIRRRGKNKVAAQNCRKRKLDQIISLADEVKEM
RDRKMRLVREREFMLIERQRVKDKFSQLYRHVFQSLRDPDGNQYHPYEYSL
QQSADGNVLLVPRNQTNPHHPRSTTMEPKTKPDPEHKE

>*Bactrocera tryoni-CncC*（XP_039957973.1）
MCWFEPILKGFVKRSRNLINSTKSKYYTYTNRGHTYVLKRKPSFEGKDGNDA
GDIDDEWAGIPFTVDNETGEYIRLPLDEILNDVLQLAEFGNINSECSNDSVAST
SKAAAAKTNNNNNINNDKTKTETGEDELNENKNVSAIISNGKDEDLAEKTLG
TITDGTTKQEQDNTEEDYIDDFDLSLSEIEVNSLPSPLFDLDDEAREELADFDI

MLQSAGAPPSPFHHHHSHQRGAGMQGYVGGGANSAFHRQASGFHHGHHQG
RMSLSRGVSMEQRLQDIASFFNPISSMGVVGGVSDMPPYPHYPTHPYSYQGA
AGIPAPPQHAQYPPPPPPHSHHHSHHAMLHANATLGDICPTQPHYGHNLGSA
VTSSMHLTNSSHEADAGAAASAGAAGGNVGAYKMEHDMMYYTNSSSDMN
HTTEGFINSILNDEDLHLMDMNDSFCRMVDVNNSTSNNSSVLGGSAGLSNGH
ASGGGGSATGSSTNGLAMNGAGATNSALAGGMTADLLGSGAGGTSAGGDR
LDASSDSAVSSMGSERVPSLSDGEWGEGSDSAQDYHQGKYGRPYDYSYNNN
NNRMNDGTRQPPVAQKKHQMFGKRFLNEQPDLGAAPTTADNGSNIGVVPPT
GTNSQLNIGPAQSIKYEYEAYPTVPGLQPGGAGGALNAEGAIGPVLSNKEQQ
AYDLKYGPAVAGAMGGYTLDFAQRPPRTPHEIVQHNHTYTLPQGTGSMPRP
QARDKKLSTSSSKSSKSINGEEDHLTRDEKRARAQNIPIAVSDIINLPMDEFNE
RLSKYDLTENQLSLIRDIRRRGKNKVAAQNCRKRKLDQILSLEDEVKEVRRR
KEQLHSDRDSLALERKRISNKFAALHRHIFQYLRDPEGNPCSTSEYTLQQAAD
GSIYLLRKSDATNGSGNISNSSSTSSSSSGSGSVSTLLSHNGMSPQQQQQQQHA
MLGTATHLQQHHHHHHAQAPQQQQQLSHIHTQQVQQGQQQQQQQQQRQH
SAHAHAHHHQTHQKD
