# Supplementary material for: Molecular Cloning and Expression Profiling of CncC in Bactrocera dorsalis Hendel
Source: Insects. 2022 Aug 30;13(9):785. doi: 10.3390/insects13090785 (PMC9503647; doi:10.3390/insects13090785)
Supplement: Supplementary file 1 [file insects-13-00785-s001.zip › Table S2 Sequences of diptera CncC for the phylogenetic analysis .pdf]

Table S2 Sequences of diptera CncC for the phylogenetic analysis.

>*Bactrocera dorsalis*
MGLPASELDVFLNSENFQDQRSVWEQNLADLRDFGDLAVNNPYAGLPLKDEP
HNNTYIDVNLDMFFPSFASAVAGGVAVKEETVGAGALNDTFPEPADIASIKTEQ
LDKEEEEFVVENEVATASSTSDEAAPKIKKETESGEDNGQAAASEEFDLSPFIS
QLDLFKEPSFEGKDGDDAGDIDDEWAGIPFTVDNETGEYIRLPLDEILNDVLQ
LAEFGNINNECSNDSVASTSKAAAAKTNNNNNNNDKTKTETGEDELNENKN
VSAIISNGKDEDLAKKTLGTITDGTTKEEHDKTEEDYIDDFDLSLSEIEVNSLPS
PLFDLDDEAREELADFDIMLQSAGAPPSPFHHHHSHQRGAGMQGYVGGGAN
SAFHRQAGGFHHGHHQGRMSLSRGVSMEQRLQDIASFFNPISSMGVVGGVSD
MPPYPHYPTHPYSYQGAAGIPAPPQHAQYPPPPPPHHHSHHAMLHANATLGDI
CPTQPHYGHNLGSAVTSSMHLTNSSHEADAGAAASAGAAGGNVGAYKMEH
DMMYYTNSSSDMNHTTEGFINSILNDEDLHLMDMNDSFCRMVDVNNSTSNN
SSVLGGSAGLSNGHASAGGSSATGSSTNGLAMNGAGATNSALAGGMTADLL
GSGAGGTSASGDRLDASSDSAVSSMGSERVPSLSDGEWGEGSDSAQDYHQG
KYGRPYDYSYNNNNNRMNDGTRQPPVAQKKHQMFGKRFLNEQPDLGAVPT
TADNGSNIGVVPPTGTNSLLNIGPAQSIKYEYEAYPTVPGLQPGGAGGALNAE
GAIGPVLSNKEQQAYDLKYGPAVAGAMGGYTLDFAQRPPRTPHEIVQHNHTY
TLPQGTGSMPRPQARDKKLSTSSSKSSKSVNGEEEHLTRDEKRARALNIPIAVS
DIINLPMDEFNERLSKYDLTENQLSLIRDIRRRGKNKVAAQNCRKRKLDQILSL
EDEVKEVRRRKEQLHSDHDSLNLERKRISNKFAALHRHIFQYLRDPEGNPCST
SEYTLQQAADGSIYLLRKSDATNGSGNISNSSSTSSSSSGSGSVSTLLSHNGMS
PQQQQQQQQHAMLGTATHLQQHHHHHHNAQAPQQQQQLSHMHTQQVQQA
QQQQQQQQQQRQHSAHAHAHHHQTHQKD

>*Drosophila melanogaster*（NP_732833.1）
MISNKKSYAMKMLQLALALSLLHYNPDYLLHRWDSQLELGTHGDGWELEM
LRTVHRLDMDHNPYGNRKGLSPRIEDLLNFDDPSLGGMANGIGGCKLPPRFN
GSTFVMNLHNTTGNSSVQTAALQDVQSTSAAATGGTMVVGTGGAPTSGGQT
SGSALGEIHIDTASLDPGNANHSPLHPTSELDTFLTPHALQDQRSIWEQNLADL
YDYNDLSLQTSPYANLPLKDGQPQPSNSSHLDLSLAALLHGFTGGSGAPLSTA
ALNDSTPHPRNLGSVTNNSAGRSDDGEESLYLGRLFGEDEDEDYEGELIGGVA
NACEVEGLTTDEPFGSNCFANEVEIGDDEEESEIAEVLYKQDVDLGFSLDQEAI
INASYASGNSAATNVKSKPEDETKSSDPSISESSGFKDTDVNAENEASAASVD
DIEKLKALEELQQDKDKNNENQLEDITNEWNGIPFTIDNETGEYIRLPLDELLN
DVLKLSEFPLQDDLSNDPVASTSQAAAAFNENQAQRIVSETGEDLLSGEGISSK
QNRNEAKNKDNDPEKADGDSFSVSDFEELQNSVGSPLFDLDEDAKKELDEM
LQSAVPSYHHPHPHHGHPHAHPHSHHHASMHHAHAHHAAAAAAAHQRAVQ
QANYGGGVGVGVGVGVGVGSGTGSAFQRQPAAGGFHHGHHQGRMPRLNRS
VSMERLQDFATYFSPIPSMVGGVSDMSPYPHHYPGYSYQASPSNGAPGTPGQ
HGQYGSGANATLQPPPPPPPPHHAAMLHHPNAALGDICPTGQPHYGHNLGSA
VTSSMHLTNSSHEADGAAAAAAAYKVEHDLMYYGNTSSDINQTDGFINSIFT
DEDLHLMDMNESFCRMVDNSTSNNSSVLGLPSSGHVSNGSGSSAQLGAGNP

HGNQANGASGGVGSMSGSAVGAGATGMTADLLASGGAGAQGGADRLDASS
DSAVSSMGSERVPSLSDGEWGEGSDSAQDYHQGKYGGPYDFSYNNNSRLSTA
TRQPPVAQKKHQLYGKRDPHKQTPSALPPTAPPAAATAVQSQSIKYEYDAGYA
SSGMASGGISEPGAMGPALSKDYHHHQPYGMGASGSAFSGDYTVRPSPRTSQ
DLVQLNHTYSLPQGSGSLPRPQARDKKPLVATKTASKGASAGNSSSVGGNSSN
LEEEHLTRDEKRARSLNIPISVPDIINLPMDEFNERLSKYDLSENQLSLIRDIRRR
GKNKVAAQNCRKRKLDQILTLEDEVNAVVKRKTQLNQDRDHLESERKRISNK
FAMLHRHVFQYLRDPEGNPCSPADYSLQQAADGSVYLLPREKSEGNNTATAA
SNAVSSASGGSLNGHVPTQAPMHSHQSHGMQAQHVVGGMSQQQQQQSRLP
PHLQQQHHLQSQQQQPGGQQQQQHRKE

>*Bactrocera latifrons*（XP_018803055.1）
MISNKKSYAMKMLQLALALSLLHVDPDNFLQQRLRNWDSQLELRDGDGWE
LEMLRAVPMVEYPYANRKTMMPLIEDLIRYDRQHDATPPQYNVTAYMLNVQ
NDGGTNQTATLPELQATTSTTTSTASSVNVAGAAALEQAANAAAAAAAASTN
ASNMGLPASELDVFLNSENFQDQRSVWEQNLADLRDFGDLAVNNPYAGLPL
KDEPHNNTYIDVNLDMFFPSFASAVAGGVAVKEETVGAGALNDTFPEPADIASI
KTEQLDKEEQEFVVENEVATSSSASDETAPKIKKETENGEDNMQATASEAFDL
SPFISQLDLFKEPSFEGKDNDDAGDIDDEWVGIPFTVDNETGEYIRLPLDEILN
DVLQLAEFGNINSECSNDSVASTSKAAAAKTNNNNDKTKTETSEDELNENKD
VSAIISNGKDEDLAKKTLDTTTDGTTKQEQDSTEKDYIEDFDLSLSEIEVYSLP
SPLFDLDDEAREELADFDKMLQSAGAAPSPFHHHHSHQRSAGMQGYVGGGA
NSAFHRQAGGFHHGHHQGRMSLSRGVSMEQRLQDIASFFNPISSMGVVGGVS
DMPPYPHYPTHPYSYQGAAGIPAPPQHAQYPPPPPPHSHHHSHHAMLHANAT
LGDICPTQPHYGHNLGSAVTSSMHLTNSSHEADAGAAASAGAAGGNVGAYK
MEHDMMYYTNSSSDMNHTTDGFINSILNDEDLHLMDMNDSFCRMVDVNNS
TSNNSSVLGGSAGLSNGHASAGGGGSATGSSTNGLAMNGAGATNSALAGGM
TADLLGSGAGGTSAGGDRLDASSDSAVSSMGSERVPSLSDGEWGEGSDSAQD
YHQGKYGRPYDYSYNNNNNNRMNDGTRQPPVAQKKHQMFGKRFLNEQPDL
GATPTTADNGSNIGVVPPTGTNSLLNIGPAQSIKYEYEAYPTVPGLQPGGAGGA
LNAEGAIGPVLSNKEQQAYDLKYGPAVAGAMGGYTLDFAQRPPRTPHEVVQH
NHTYTLPQGTGSVPRPQARDKKLSTSSSKSSKSVNGEEEHLTRDEKRARALNI
PIAVSDIINLPMDEFNERLSKYDLTENQLSLIRDIRRRGKNKVAAQNCRKRKLD
QILSLEDEVKEVRRRKEQLHSDRDSLALERKRISNKFAALHRHIFQYLRDPEG
NPCSTSEYTLQQAADGSIYLLRKSDATNGSGNISNSSNTSSSSSGGGSVSTLLS
HNGMSPQQQQQQQHAMLGAATHLQQHHHHQHAQAPQQQQQLSHMHTQQV
QQAQQQQQQQQQQRQHSAHAHAHHHQTHQKD

>*Bactrocera oleae*（XP_036217502.1）
MISNKKSYAMKMLQLALALSLLHVDPDNFLQQRLRNWDSQLELRDGDGWE
LEMLRAVPMVEYPYANRKTMMPLIEDLIRYDRQHDATPPQYNVTAYMLNVQ
NDGGTNQTATLPELQATTSSTTSAASSVNVAGAAALEQAANAAAAAAAASTN
ASNMGLPASELDVFLNSENFQDQRSVWEQNLADLRDFGDLAVNNPYAGLPL
KDEPYNNTYIDVNLDMFFPSFASAAAAGVAVKEETVGAGALNDTFPEPADIAS

IKTEQLDKEEQELALENEVATSSSSSDEAAPKIKKEKESGEHNTQAALSEEVDL
SPFISQLDLIKEPSFEGKDVGDDAGDIDDEWAGIPFTVDNETGEYIRLPLDEILN
DVLQLAEFGNINSECSNDSVASTSKAAAAKTNNNNNNNDKTKSETEEDEIND
NSSNNNNNASAIISNGKDEDLTKKTLGTTTDVTTKHKEDNTEEDYIDDFDLSL
SEIEVNSLPSPLFDLDDEAREELADFDIMLQSAGAPPSPFHHHHPHQRGAGMQ
GYVGGGANSAFHRQATGFHHGHHQGRMSLSRGVSMEQRLQDIASFFNPISSM
GVVGGVSDMPPYPHYPTHPYSYQGAAGIPAPPQHAQYPPPPPPHPHHHSHHA
MLHANATLGDICPTQPHYGHNLGSAVTSSMHLTNSSHEADGGAAAAAGAAG
GNVGAYKMEHDMMYYTNSSSDMNHTTEGFINSILNDEDLHLMDMNDSFCR
MVDVNNSTSNNSSVLGGSAGLSNGHASASGGSATGSSANGLAMNGAGATNS
ALAGGMTADLLGSGAGGTSAGGDRLDASSDSAVSSMGSERVPSLSDGEWGE
GSDSAQDYHQGKYGRPYDYSYNNNNNRMNDGTRQPPVAQKKHQMFGKRFL
NEQPGLGATGTTADNGSNVGVVPPTGTNSLLNIGPAQSIKYEYEAYPTVPGLQ
PGGAGGTLNAEGAMGPVLSNKEQQAYDLKYGPAVAGAIGGYSLDFAQRPPRT
PHEVVQHNHTYTLPQGTGSMPRPQARDKKLSISSSKSSKSLNGEEEHLTRDEK
RARAMNVPIPVSDIINLPMDEFNERLSKYDLTENQLSLIRDIRRRGKNKVAAQ
NCRKRKLDQILSLEDEVKEVRRRKEQLFSDRDSLTLERKRISNKFAALHRHIFQ
YLRDPEGNPCSTSEYTLQQAADGSIYLLRKSDATNGSGNISNSSSTSSSSSSSS
VSTLLSHNGMSPQQQQQQQPQQHSMLGAATHLQQQQQSLHHHHHHTQAQ
QQQQQLSHMHTQQVQQAQQQQQQQRQHSAHAQAHHHQTHQKD

>*Zeugodacus cucurbitae*（XP_011186373.1）
MISNKKSYAMKMLQLALALSLLHVDPDNFLQQRLRNWDSQLELRDGDGWE
LEMLRAVPMVEYPYANRKTMMPLIEDLIRYDRQHDATPPQYNVTAYMLNVQ
NDGGTNQTATLPELQATTSSTSSAGGSVNVAGAAALEQAANAAAAAAAAST
NASNMGLPASELDVFLNSENFQDQRSVWEQNLADLRDFGDLAVNNPYAGLP
LKDEPNNNTYIDVNLDMFFPSFASAAAAGAVVKEESVGAGALNDTFPEPADIA
SIKTEQLDKEETEFALDNEVATSSSASSDEAAPKIKKEPESADDNAQAASTEEV
DLSPFISQMDLLKEPSFEGKDVGDDAGDDEWAGIPFTVDNETGEYIRLPLDEIL
NDVLQLAEFGNINSECSNDSVASTSKAAAAKTNNNNNNNDKTKSETGEDEIND
NNNNNNSAIIDNGKEEDLLKNTFGTTTDNNTKHEQQDITEDDYIDDFDLSLSEI
EVNSLPSPLFDLDDEAREELADFDIMLQSAAAPPPPFHHHHPHQRGAGMQGY
VGSGANSAFHRQASGFHHGHHQGRMSLSRGVSMEQRLQDIASFFNPISSMGV
VGGVSDMPPYPHYPTHPYSYQSAAGIPAPPQHAQYPPPPPPHPHHHSHHAML
HANATLGDICPTQPHYGHNLGSAVTSSMHLTNSSHDTDGGAAAAAGAAGSN
VGGYKMEHDMMYYTNSSSDMNHTTEGFINSILNDEDLHLMDMNDSFCRMV
DVNNSTSNNSSVLGGSGGLTNGHAGVGGGSATGSTTNGHAMSGAGATNSAL
AGGMTADLLGSGAAGASAGGDRLDASSDSAVSSMGSERVPSLSDGEWGEGS
DSAQDYHQGKYGRPYDYSYNNNNNRMNDGTRQPPVAQKKHQMFGKRFLNE
QPGLGATGTNADNGSNVGVVPPAGTNSLLSIGPAQSIKYEYEAYPTVPGLQQG
GAGSALNAEGAMGPVLSNKEQQAYDLKYGPAVAGAMGGYSLDFAQRPPRTP
HEVVQHNHTYTLPQGTGSMPRPQARDKKLSISSSKSSKSMSEEEHLTRDEKRA
RAMNIPIAVSDIINLPMDEFNERLSKYDLTENQLSLIRDIRRRGKNKVAAQNCR
KRKLDQILSLEDEVKEVRRRKDQLYSDRDSLTLERKRISNKFAALHRHIFQYL

RDPEGNPCTTSEYTLQQAADGSIYLLRKSDAASGSGNISNSSSTSSSSSSSSSNV
STLLSHNGLSPQQQQQQQQHSMLGAATHLQQQQQALHHHHQQHAQAQAQQ
QQLSHMHPQQVAQQQQQQQQQQQQQRQHSAHAHAHHHQTHQKD

>*Bactrocera tryoni*（XP_039957973.1）
MCWFEPILKGFVKRSRNLINSTKSKYYTYTNRGHTYVLKRKPSFEGKDGNDA
GDIDDEWAGIPFTVDNETGEYIRLPLDEILNDVLQLAEFGNINSECSNDSVASTS
KAAAAKTNNNNNINNDKTKTETGEDELNENKNVSAIISNGKDEDLAEKTLGT
ITDGTTKQEQDNTEEDYIDDFDLSLSEIEVNSLPSPLFDLDDEAREELADFDIM
LQSAGAPPSPFHHHHSHQRGAGMQGYVGGGANSAFHRQASGFHHGHHQGR
MSLSRGVSMEQRLQDIASFFNPISSMGVVGGVSDMPPYPHYPTHPYSYQGAA
GIPAPPQHAQYPPPPPPHSHHHSHHAMLHANATLGDICPTQPHYGHNLGSAVT
SSMHLTNSSHEADAGAAASAGAAGGNVGAYKMEHDMMYYTNSSSDMNHT
TEGFINSILNDEDLHLMDMNDSFCRMVDVNNSTSNNSSVLGGSAGLSNGHAS
GGGGSATGSSTNGLAMNGAGATNSALAGGMTADLLGSGAGGTSAGGDRLD
ASSDSAVSSMGSERVPSLSDGEWGEGSDSAQDYHQGKYGRPYDYSYNNNNN
RMNDGTRQPPVAQKKHQMFGKRFLNEQPDLGAAPTTADNGSNIGVVPPTGT
NSQLNIGPAQSIKYEYEAYPTVPGLQPGGAGGALNAEGAIGPVLSNKEQQAYD
LKYGPAVAGAMGGYTLDFAQRPPRTPHEIVQHNHTYTLPQGTGSMPRPQARD
KKLSTSSSKSSKSINGEEDHLTRDEKRARAQNIPIAVSDIINLPMDEFNERLSKY
DLTENQLSLIRDIRRRGKNKVAAQNCRKRKLDQILSLEDEVKEVRRRKEQLHS
DRDSLALERKRISNKFAALHRHIFQYLRDPEGNPCSTSEYTLQQAADGSIYLLR
KSDATNGSGNISNSSSTSSSSSGSGSVSTLLSHNGMSPQQQQQQQHAMLGTAT
HLQQHHHHHHAQAPQQQQQLSHIHTQQVQQGQQQQQQQQQRQHSAHAHA
HHHQTHQKD

>*Ceratitis capitata*（XP_012159075.1）
MISNKKCYAMKMLQIALALSLLHVDPEQRLRNWDSQLELRDGDGWELEMLR
AVPMVEYPYANRKTMMPLIEDLIRYDRQHDPTPPQYNVTAYMLNVQNDGGT
NQTATLPEIQATSSGTTNPASNVAVAGATALEQAANAAAAAAAASTNASNMGL
PASELDVFLNSENFQDQRSVWEQNLADLRDFGDLAINNPYAGLPLKDEPYNN
TYIDVNLDMFLPSFASAAAAAAAAANDVKQENVAAGALNDTFPEPADVASIK
TEQIDKEEAEEQEFVLNNEASSSGVSSAETVFTTTPTSQAAIIKKEPIESSGDDE
AQTEMEEVDLSPFFSQLDIMKEESEIIDVLWKQDVDLGYSLTPAHLINTTGNN
GSAGAVSTDDEIEKLKALEALKLDKPNFDGKDGGDDAGVPDIDDEWAGIPFT
VDNETGEYIRLPLDEILNDVLQLAEFGNLNNEPNNDDSYASTSKAAAVKNDD
DKRITKSETSEDEGINDDNITSARLVNGKEETEKDSNKKTLATSTTDKDTKLER
ESINENDYIDDFDLSLSEIEVNSLPSPLFDLDDEAREELADFDMMLQSAAAPPP
TFHHHHPHSHQRGAGMQGYVGGGANSAFHRQASGFHHGHHQGRMSLSRGV
SMEQRLQDIASFFNPISGMGVVGGVSDMPPYPHYPTHPYSYQSAAGIPAPPPQ
HAQYPPPPPPHPHHPHSHHAMLHANATLGDICTPTQPHYGHNLGSAVTSSMH
LTNASHDADGGASASAGAAGGNVGAYKMEHDMMYYTNASSDMNHTTEGFI
NSILNDEDLQLMDMNDSFCRMVDVNNSTSNNSSVLGGGLAAGHASTGGGSA
TGSIANGLTMSGAGAANSALTGGMTTDLLSSGAGATSAGGDRLDASSDSAVS

SMGSERVPSLSDGEWGEGSDSAQDYHQGKYGRPYDYSYNNNSNNRMNDGT
RQPPVAQKKHQMFGKRFLNEQPGLGAVTARPDNNSNAGGGVNVLPPTGANN
LLNIGPAQSIKYEYEPYPTVPGLQQGGSGGVLNADGAMGPALSSKEQQAYDL
KYGPAVAGAIGGYSLDFAQRPPRTPHEVVQHNHTYTLPQGTGSMPRPQARDK
KLSISSSKSSKSMNGEEEHLTRDEKRARALNIPISVSDIINLPMDEFNERLSKYD
LTENQLSLIRDIRRRGKNKVAAQNCRKRKLDQILSLEDEVKAVRSRKEQLYAD
RDSLTLERKRISNKFAALHRHIFQYLRDPEGNPCSTSEYSLQQAADGSIYLLRK
SDATNTGGNASNSSSTSSSSSGSSSSAGGGGGGGAAAAAEGVNGHTMRHNG
VSPQQHAMLGAATHLQQQQQQTLHHQHHHAQTQQQLSHMHPQQAQQQ
QQQQQQQQRQHSAHAHAHHHQTQQKD

>*Musca domestica*（XP_011293624.1）
MISNKKSYAMKMLQLALALSLLHVNPDDYLQQRLRNWDSQLELRDGDGWE
LEMLRAVPMVEYPYANRKSMMPLIEDLMLYDHSLANTQYNVTAYLLNVSNE
SANQTAAMQEMQAIPPPSSATTTGGSSSNSPTSSSSPSSGGSLTLENVRNGTTAT
VSSIVHAAGSSNSSNLHNLPTPEIELFLNSESMQDQRSVWEQNLADLNDFNDL
PIMNSHYGNLPLKDGQQQDPSGHFDLPHLDVFLPTFGSSANALNSEAEGAVAL
NGSFPNPDDAVGIKLEQLDDEDDKALLVGVMDTRAATCSTLSPDPTDIAVDAC
IKKEEPEEEVVGQTVKEEEVDLSPFIANMELTKEESEIVEVLWKQDVDLGFSLT
EAQLAELAGSSSATENAVNSEDEIEKLKALEALKLDNPKYDSKDVDDEDSNQ
LASNEWTGIPFTIDNETGEYIRLPLDDILNDVIQFAELAGGENDEDDTTGASTS
KAAAAAANKKILKTDNEDTDNNDDNDPTDNHSAKKNPSLDKTLKSEGGDYI
PDDLENLHTEKQLSKGNKSSDTTKGDANEEDNDDTNNDHDDNDLPQSEIEVS
SILSPLFDLDDEAKEELADLSMMLHSSAPFAPHHHHPHHPHHRGAAGGVVGG
AQNYGSVNSAFHRQPSSGGFHHGHHQGRMPLSRGVSMEQRLQDIASLFNPIP
SMGVVGGVSEMSPYPHYPTHPYTYQSAPQHAQYPPPPPHPHHHHHAAAAAM
LHPNASLGDLCSTQPHYGHNLGSAVSSSMHLTNSTHDSDVTGVGAGAVGGAY
KMEHDMMYYSNASSDMNHTTDGFINSILNDEDLHLMDMNVNDSFCRMVDV
NNSTSNNSSVLGLPTGTGHASGAASGGSTAGSTTSGISMSAASNVAASAGAPV
MTNDLMNATGAATQTVAGGTDRLDASSDSAVSSMGSERVPSLSDGEWGEGS
DSAQDYHQGKYGGPYDYSYNNSATNRLNDGSRQPPVAQKKHHMFGKRFMN
EQPAAANLGAAGSGANGPAAGSNNVGALVHGGPAHSIKYEYEAYPPVAGLHE
NMNGHGLDNGAMGGPALSKDHHGYGPGAYSLDFSVRTPRTPHELVQHNHTY
TLPQGTGPLPRPQQRDKKLSTSSSTKSAKSSHNSSGSGGEEETYMTRDEKRAR
AMNIPIPVCDIINLPMDEFNERLSKYDLSENQLSLIRDIRRRGKNKVAAQNCRK
RKLDQILSLEDEVKQVRRRKDELNAQRDRLFTERKRVSNKFSALHRHIFQYLR
DPEGNPCSPSEYSLQQAADGSIYLLPRDAANKNDTNSAVGLLNGGAGIGSPLN
GHSQSMPTAVGHHHSAHPAPGAGGGHNLHQAPHQQTVLHTAAGSLAHSHVQ
HHHQHHHHQQQHNQTSQPQTTTHAHGHHTTTHGGSQQQQPQKD

>*Teleopsis dalmanni*（XP_037948765.1）
MRSNKKSYAIKILQIALALSLLQGDPDIYLQQRLRSWDSQLELRDGDGWELEL
LRAVPMVEYPYANRKTMMPLIEDLIRYDRQREKHNHQNITAYLLVHNDLATN
QTAAIQELQATPNAQTVSNPSTGAAGLEHVNAANLASTELNVNASRLSPIPEL

DMFLNLDTAFDPRPIWEQNLANLSDYGDLSGLNHYNGLPLKDEPSSSTYLDV
YLPNFSDDSLKDIKTEGATALNDTFPNPGDLTSIKNEHSDNNDLNELEALNNL
ASCSSNSLSQRCIKKEPVDSEKDTNDSDPFLTTEESEIVEVLWKQDVDLGFSLT
SELQLNSNTGESDIDLLKTKQLDSSQQDNEDINKLKALEELKLDKLKYDSKDN
NHDEDIGDEWAGIPFIIDNETGEYIRLPLDELLHDVIQLSELAGKDNEVESDPIA
STSKAAGVQSNNKQLNKLDFDGKNIEDKDNVDNNNKNNTNNNNDSNNYKN
DGELSNEDDLPQSEIEVGLLPSPLFDLDDEAKEELVEDLDRMIQSATPAFHHPH
HHHPHRPTAAGIQNYAVGSGANSAFHRQATGFHHGHHHQGRVSLSRGVSME
QRLQDIANFFNPIPSMGVVGGVSDMGGYPHYPTHPYSYQGATGITAPPQHSQY
PPPPPHHHGHHTMLHPNATLGDLCSNQPHYGHNLGSAVTSSMHLTNASHDAD
LGAGATGGSSNAGPYKMEHDLMYYNVSSDINHTTDGFINSILNDEDLHLMD
MNVTDNFCRMVDINNSTSNNSSVLGLASGHTSTGGGSGTNSLSMNGVTNSA
GSGMTTDLINQGGQVGDRLDASSDSAVSSMGSERVPSLSDGEWGEGSDSAQD
YHQGKYGGPYDYSYSNNNRLNDGTRQPPVAQKKHQMFGKRYMHDQPSITG
ATAGVNSTAPVNGMTPSNPLLHGPAQSIKYEYEPYTATGLHQNSNGIGLGEGA
MVPVLNKDQQTYDMKYSNGTLGTYSIDYSIRQPRTTQEIVHHNHSYTLPQGT
GSLPRPQLRDKKLSTSSSSIKSSKSVSSSEDEHLTRDEKRARALNIPIAVIDIINLP
MDEFNERLSKYDLTENQLSLIRDIRRRGKNKVAAQNCRKRKLDQIVSLEDEV
KEVRKRKDELQSDLEGLSSARKHISNKFAALHRHIFQYLRDSEGNPCSPSEYSL
QQAADGSIFLLPRDSSNKTDAVNGIAATSSNNNNSNNSINGGGHLNNSTTTPL
QHPHVQVAATHQQHSHQHNHNLTQPLSHHSVQLQHQQQQQQQQQQQHHQ
HAHHHHQSHRKD

>*Sarcophaga bullata*（TMW53478.1）
MISNKKSYAMKMLQLALALSLLHVDPDNYLQQRLRNWDSQLELRDGDGWE
LEMLRAVPMVEYPYANRKTMMPLIEDLIRFDRQNDHLATQYNITAYLLNVSN
ESSNQTAALQEMQQLPSTPTTNSAASGSTTTATASASLDPSTVTVSSVVTAPGA
TNASHLNNFPTSEIELFLNSENFQDQRSVWEQNLADLNDFNDLPIMNSQYGNL
PLKDGQQDNNLFDLQLDAFLPTFGSSAATAPTSVEGAVALNDTFPNPDEGAGI
KLEQLDDEDDKALLIGVIDTRTVSALSTDEQALAEFNVKKEPEDSNSDNDNTT
ANNSEVDLSPFINTVELTKEESEIVEVLWKQDVDLGYSLTPAQLAEADASAKA
LQQQSTVNDDDIEKLKALEALKLDNGKYDPKDIDNDDHLGNEWAGIPFTIDN
ETGEYIRLPLDEILNDVIQFAELAGEDEDTGASTSKAAAANSKNHLKSKKEEE
DNNQDSNSTASATKTETDFDEEFKSEGDFKDDLNGEDDKKKKKKKNKKKYN
NGIDDEKDNNKSNDNNNKHKINSDDEVDNDDEDDDDDEDSDLPQSEIEVNSL
PSPLFDLDDEAKEELADLSMMLHSSAPFAPHHHPHHPHHPHAHARAGGAGA
AAGGVGAVQNYGSVNSAFHRQPSSGGFHPGHHQGRMPLSRTVSMEHRFQDI
ASLFNPMGVVGGVSDISPYSHYPTHPYTYQTGPQHAQYPPPPPPPHPHHHHA
AAMLHPNASLGDLCSTQPHYGHNLGSAVSSSMHLTNSSHETDVAGVGAGAV
GGSAYKMEHDMMYYSNASSDMNHTTDGFINSILNDEDLHLMDMNVNDSFC
RMVDVNNSTSNNSSVLGIPSGHGNTASGSASGSTSGLSMSSSNIAGAAGASG
MTSDLMNSVATQGGTDRLDASSDSAVSSMGSERVPSLSDGEWGEGSDSAQDY
HQGKYGGPYDYSYNNTTNNRLNDGTRQPPVAQKKHHMFGKRFLNEQPGVN
AANANPAVSTNDVINGPAHSIKYEYEPYPSITVINENSLVNGLEGGAMGGPTLS

KEHHAYGGAYSLDFSMRTPRTPHEIVQHNHTYTLPQGTGSLPRPQQRDKKTSI
SSSSTKSSKSAHNSSGSGGEEENYLTRDEKRARAMNIPIPVTDIINLPMDEFNE
RLSKYDLSENQLSLIRDIRRRGKNKVAAQNCRKRKLDQILSLEDEVKQVRRRK
EDLQAQRERLFNERKRVSNKFGALHRHIFQYLRDSDGNPCSPAEYSLQQAAD
GSIYLLPRDTTIKNETNGVLHGSHTTNGSSQPHHPQMSQNSHVAALHRHHLPQ
QASTAAVTASSMAHNHAVQHHHHQQQQHHHQPATQGQPTHNGHHQPTQH
GGTAANQQKD

>*Lucilia cuprina*（XP_046805387.1）
MISNKKYAMKMLQLALALSLLNVDPDNYLQQRLRNWDSQLELRDGDGWEL
EMLRAVPMVEYPYANRKTMMPLIEDLIRFDRQSDHLANQYNVTAYLLNVSNE
NSNQTAAMQEMQAIPATSSNNNPSSGSSATTTSSSALDSSTATVSSVVTAPGAT
NASHISNFDNSEMELFLNAENFQDQRTVWEQNLADLNDFNDLPILNSHYGNL
PLKDGQQDNNLFDLQLDAFLPTIRSSASTSESEGAVALNDTFPNPDEEVAIKLE
QLDDEDDKALLMGVMDTRAVTALSTDEVAIKQVSVKKEEPDESAEDSGIEAV
TDSEVDLSPFINTVELTKEESEIVEVLWKQDVDLGYSLTPAQLADAGTSAKAL
QSSNANDDDIEKLKALESLKLDNSKYDPKDIDDNDDDNHLGDEWAGIPFTID
NETGEYIRLPLDDILNDVIQFAELAGEEEDDTGASTSKAAAANNNNNSNLLKS
EKEEQEDEQKSTATIKTTAATKTETDFGKDYKSEGDLKDDLNSDDNNKRKNG
TGKEDKNKAGSDEDDDDNDSDLPQSEIEVSSLPSPLFDLDDEAKEELADLSM
MLHSSAPFAPHHHHHPHAHHARAGGAVGGVGGVQNYGGVNSAFHRQPSSG
GFHHGHHQGRMPSLSRAVSMEHRFQDIASLFNPMGVVGGVSEMSPYPHYPSH
PYSYQSGPQHAQYPPPPPHPHHHHHAMLHPNASLGDLCSSQPHYGHNLGSAV
SSSMHLTNSTHESDVSGVGAGAVGGSAAANIGAYKMEHDMMYYSNASSDM
NHTTDGFINSILNDEDLHLMDMNVNDSFCRMVDVNNSTSNNSSVLGISSGHA
NAATGSANGSTSGISMSGTNVGAAGGAAGMTSDLMNSAGAQGGTDRLDASS
DSAVSSMGSERVPSLSDGEWGEGSDSAQDYHQGKYGGPYDYSYNNSTSNNR
LNDGTRQPPVAQKKHHMFGKRFLNEQPAVNAANGANPAATGVNGVINGPAH
SIKYEYEPYPPVAGLHENMDNGLQGGAMGGPTLSKEHHTYGGAYSLDFSVRT
PRTAHEIVQHNHTYTLPQGSGSLPRPQQRDKKTSTSSSSTKSSKSAHNSSGSGG
EEDNYLTRDEKRARAMNIPIPVTDIINLPMDEFNERLSKYDLSENQLSLIRDIRR
RGKNKVAAQNCRKRKLDQILSLEDEVKQVRRRKEDLQAQRDRLFNERKRVS
NKFGALHRHIFQYLRDPEGNPCSPSEYSLQQAADGSIYLLPRDAANKNDTSSG
VLQHGSTANGSSQPTHHQQMSQTHTNAHVAGGHHHPHLQQQTTTAATAAAA
SLAHSHVQPHHHHHQQTQQHLHQPGQTTHPHPQTQHNGGGGTAHQQKD

>*Glossina fuscipes*（XP_037893857.1）
MKTYIYKESEIVEVLWKQDVDLGYSLTSEQSSVNVPSSTSAKAANSDDIEKLK
ALEELKLDGTKYDTKNPINENDQLDDDWAGIPFTIDNETGEYIRLPLDEILNDV
IQFAELAGETGEINESSNGASTSAAAAAIVNNQNSIKLKSERDVEQHSDELTGN
NNNSAFKSTTTTTTTLKTIKSEVDVKKEKSIEVSQKDKKNNNNKTESEASTKN
KGKVEQDGDDDDDDDDDLTQSEVDSLPSPLFDLDDEAKEELADLDMMLHSS
APFAPHHHPHPHAHHPHHHHHHHHHHRATGTTGSQNYGGVNSAFHRQPSST
GGFHHGHHQSRMPLSRGVSVEQRLQDIASLFNPMGVVGGVSEMSPYSHYPT

HHYSYQSATQHAQYPPPPPPPPPPHPHHHHHATVLHPNASLGELCSAQPHYGH
NLGSAVSSSMHLTNSTHDADISGVGAVGGVSNVGAYKMEHDMLYYSNATSD
MNHTTDGFINSILNDEDLHLMDMNVNVNDNFCRMVDVNNSTSNNSSVLGLS
GGHASATGAGPVSSSGLSMSGNNVNAAPGGAATMTSELMVTGSGQTSGDRL
DASSDSAVSSMGSERVSSLSDGEWGEGSDSAQDYHPGKYGGPYDYSYNSSTT
NSRLHDGTRQPPVAQKKHHMFGKRFLNEQPPGSNVAAVATASSAANANAGVL
HGPAQSIKYEYEPYPPAVAGLHQNMGNGIESGAIAPILTNKEHHSYGGGAYSLD
FSLRPPRTPQELIQHNHTYTLPQGSGSIPRPQVRDKKSISSSTSIKSIKSTNSSGS
GGEDDVYMTRDEKRARAMNIPIPVPDIINLPMDEFNERLSKYDLSENQLSLIR
DIRRRGKNKVAAQNCRKRKLDQILSLEDEVKQVRRRKDELQQQRDRFLTEHK
RVSNKFSALHRHIFQYLRDADGNPCSTSEYSLQQASDGSIYLLPRDTSSNNKN
EALTNMNVNSGILSSTVSANNNNNNNSILAPHQIQTSMSATNTHHQQQHTTQVT
MAHNHLQQQQQQQQHHIHPHHQTQQNQVTATGTVAQTQQSTQTTGNGISGA
GGGGHYHQPQKD

>*Rhagoletis zephyria*（XP_017484642.1）
MVDVNNSTSNNSSVLGSGLTSGHASAGGSSATGSTSNGLAMSAAGAANTALS
GGMTTDLLNSGAGTTAGGDRLDASSDSAVSSMGSERVPSLSDGEWGEGSDSA
QDYHQGKYGGPYDYSYNNNNNRLNDGSRQPPVAQKKHQMFGKRFLNEQPGL
LGAAGATAGNNNNAGVAPPSSANSLLNVGPAPSIKYEYEPYPTVPVLQPGGTI
EGALRADGALGPSLSSKEQQAYELKYGPVAADAIGDYTLDFAQRPPRTPHEV
VQHNHTYTLPQGTGSLPRPQARDKKLSTSSSKSSKSLNAEDEHLTRDEKRAR
AMNIPIAVSDIINLPMDEFNERLSKYDLTENQLSLIRDIRRRGKNKVAAQNCRK
RKLDQILSLEDEVKEVRRRKEQLYSDRDSLALERKRISNKFAALHRHIFQYLR
DPEGNPCSTSEYTLQQAADGSIYLLRKSDATNSGGNASNSSSTSSSSSSSSSG
VSGLLNHNGMPPQQQHQHSMLSSATHLQQQQQSQSLHHHHHHQAQSQQQQ
LAHMHTQQAQQSQQQQQQQQRQHSTHAHAHHHQTHQKD

>*Drosophila elegans*（XP_017131181.1）
MCLNEEYKYMHIYLAESEIAEVLYKQDVDLGFSLDQEAIINASYASGNSGATN
AKSKLEDEAKSSDPSMSDTGASKDPNVSAENEASSASAEDLEKLKALEELRQ
DKEKDNENPLEDITNEWNGIPFTIDNETGEYIRLPLDELLNDVLKLSEFPLEDE
LSNDPVPSTSQAAAALNENQALRIVSETGEDFLSGEGIPNKQRKVGDKDKDT
DPEKGEGDSFSVSDFEDLQNSVGSPLFDLDEDAKKELDEMLQSTAPPYHHPHP
HHGHPHAHPHPHSHHHATMHHAHAHHAAAAAAAHQRAVQQANYGGGVGV
GVGVGVGVGSGTGSAFQRQPAAGGFHHGHHQGRMPRLNRSVSMERLQDFAT
YFSPIPSMVGGVSEMSPYPHHYPSYSYQASPSNGAPAPGQHGQYGSGAAAPL
QPPPPPPPPHHAAMLHHPNAALGDICPTGQPHYGHNLGSAVTSSMHLTNSSHE
ADSAAAAAAAYKVEHDLMYYGNTSSDINQTDGFINSIFADEDLHLMDMNESF
CRMVDNSTSNNSSVLGLPSSGHVSNGSGSSAQLAAGNPHGNQVNGAASGVG
PMSGSVVGTGAAGMTADLLASGGAGAQGGTDRLDASSDSAVSSMGSERVPS
LSDGEWGEGSDSAQDYHQGKYGGPYDFSYNNNSRLSTATRQPPVAQKKHQL
YGKRDPHKQAPSAVPPTAPTAASTAVQSQSIKYEYDAGYASSGMASGGVASEP
GAMGPALTKDYHHHQAYGMGASGSTFSGDYTVRPSPRTSQDLVQLNHTYSLP

QGSGSLPRPQVRDKKPLVATKTASKGTSAGSSSSGAGGSSGNLEDEHLTRDEK
RARSLNIPISVHDIINLPMDEFNERLSKYDLSENQLSLIRDIRRRGKNKVAAQN
CRKRKLDQILTLEDEVHAVVKRKSQLNQERDHLDSERKRISNKFAMLHRHVF
QYLRDPEGNPCSPSDYSLQQAADGSVYLLPREKPEGNSTATSASSAVSAVAGS
LSGHVPSQAPMHGHHSQHGMQAQHVGSGMSQQQQQQQSRLPPHLQQQQQQ
QHHLQSQQQQPGGQQQQHRKE

>*Drosophila kikkawai*（XP_017017474.1）
MCLNEEYKYMHIYLAESEIAEVLYKQDVDLGFSLDQEKIINASNASGNTGASS
AKSKYGDESKSSDPSMSDSAIKDANDNAEKEAGEASVDDIEKLKALEELQQD
KDKDNENPLEDITNEWNGIPFTIDNETGEYIRLPLDELLNDVLKLSEFPLEDEL
SNDPVASTSQAAAALNENQAQRIVSETGEDFLSGEGVPSKQRRIGAEDKDND
QDKADGDSFSVSDFEDLQNSVGSPLFDLDEDAKKELDEMLQSTAPPYHHPHP
HHGHPHAHPHPHSHHHATMHHAHAHHAAAAAAAHQRAVQQANYGGGVGV
GVGVGVGSGTGSAFQRQPAAGGFHHGHHQGRMPRLNRSVSMERLQDFATYF
SPIPSMVGGVSDMSPYPHHYPGYSYQASPSNGGNPSAPGQHGQYGGGAAAPL
QPPPPPPPHHAAMLHHPNAALGDICPTGQPHYGHNLGSAVTSSMHLTNSSHEA
DGAAAAAAAYKVEHDLMYYGNTSSDMNQADGFMNSIFTDEDLHLMDMNES
FCRMVDNSTSNNSSVLGLPSSGHVSNGSGSSAQLAAGNPHGNQANGAAGGV
GPMSGTGAAGMTTDLLASGGAGAQGGTDRLDASSDSAVSSMGSERVPSLSD
GEWGEGSDSAQDYHQGKYGGPYDFSYNNNSRLTTATRQPPVAQKKHQLYGK
RDPHKQTPSALPPTAPPATATAVQSQSIKYEYDAGYSSSGMASGGISEPGAMGP
ALSKDYNHHQAYGMGASGSGFSGDYTMRPSPRTSQDLVQLNHTYSLPQGSGS
LPRPQARDKKTVVATKTATKGTNPGSSNSSAGGGSSSSSNTLEDEHLTRDEKR
ARSLNIPIPVQDIINLPMDEFNERLSKYDLSENQLSLIRDIRRRGKNKVAAQNC
RKRKLDQILTLEDEVNAVVKRKAQLNADRDHLEGERKRISNKFALLHRHVFQ
YLRDPEGNPCSPADYSLQQAADGSVYLLPRDKTEGNSTATAASNAVSSASGVS
LNGHVPAQPSMHGHHGQHGMQAQHVGGGLPQQQQQSRLPPHLQQQQQHHL
QSQQQPGGQQQQQHRKE

>*Hermetia illucens*（XP_037902807.1）
MANCRFIVKESEIVEVLWKQDVDLGFSLAPPKPDKSAPQPGSSKNSEDEAEKL
KALQDLKDENNESKLNDEGKDDLDDEWAGIPFTIDNETGEYIRLPLQLDELLQ
DVLQLADLNPDEQENHQEPKPNPDVKLITEEKPSTSGSGASSSSSNVVSTSSLS
DEATIVEPPQSEVEVASAQSELFNLDNADALDELDMMIQSAGSFQHHRPGQN
YGTNSVFRQSGFHHHHHQSRVPLTRGVSMEQRLQDIANLFNPLPAMGVGMG
VSEMSPYSHYPTHYPYQGGPAIPQHGQFPHPSHHPVLHNASLADLGATQPHY
GHNLGSAVSSSMHLTNSSHETDSGASGYKMEHDMMYYSNTSSELNHTTDGFI
NSILNDEDLQLMDMGVSEGFCTMRMLEGNTTSNNSSVLGSSGASHGSHMLS
MPTNLGNLSNEVIGASTGDRLDASSDSAVSSMGSERVPSLSDGEWGDAGSDS
AQDYHQGKYGGPYDYSYNGRLGGDSSRQQPVAQKKHQMFGKRYFQEQPNIP
SLPQPSSTTQPPSDHLPPVPVKYEYDAYGMHPSGAASIASSLEGAVGPIVNKDH
VPLMTNAEMKYACSLDFSRPPRGSGPAHDLISHNHTYTLPQGSGSVPRPQIRD
KKQKRSEDEHMTRDEKRARALNIPISVNDIINLPMDEFNERLSKYDLNETQLS

LIRDIRRRGKNKVAAQNCRKRKLDQILSLEDEVKEVRRRKERLFQERDIILAER
KRIASKFATLHRHVFQYLRDSDGNPCSPSQYSLQQAADGSVYLLRRENNKPE
NNTTTNHSGHHHHSSSQGPQPPSSSSQHQKD

>*Aedes aegypti*（XP_021693907.1）
MAALEYEDLPRMQSLTPEPDDTGSLKDGMGAPEFEQQQQQATTQVVVIGGD
SSCNLADDCCNDKPNSCDHDEVGYHSPLYNYSKYNLKEMDLIEVLWKQDVD
LGFTLTPLTVPSGASGGATSGMATKGATSAEDSSDDLEKLKVLLEIKHDDDKK
KDNADKQAEENPWAGMSYTVDIETGEYVLNATDGSEATLPPPLADLFLEEGL
NLPELINDTVIEETEEPAKKKTTAEQEEEEESEVKRIERELESSADELCQGAAAS
VSATSGSTSASPCDLNICSEQQATATSSVVDREQQDSPNHPEAAGAASPDDLED
ILCDMMIQTSSSHFQHPRQPMQNYGHGMHGSLRQGGYHHHHHHHHHQSRVP
LSRAVSMEQRFQDLANLLSFPPGMGVGMGVGEMPPAHPHPHYPPHYSYQAN
GAIPQHGQYHSHAVLQNASLADIGPTQPYYAPNLGSAVATSMHLTNSTSETDA
GATGYKMDHEMMYYSNTSSEMNHTDGFLNSILDDDLQLMDIAVNEGMYTM
RMLDHNATSSNSSVLGGSLGGSAAAVAAAAAAGLNGPAHLGGLMSSASAAV
SSGAMQTSLNGSTGTHGATGGTTSGDRLDASSDSAVSSMGSERVPSLSDGEW
GDGGSDSAQEYHNKYGGPFDYSYSGSNRLGDGTRQPPVAQKKHHMFAKRYF
QEQNTSIPSLPSATNPSATGPTVDPQSQLNASIPIKYEFDYMNPASLSHLEGAVG
PVTKQEDQTSAHNNPLSSVDMKYPYSLDFSRQNPASAPAARSHHHDVIHHNH
TYTLPHNSGANPKPQTRDKRIRKAEEEHLTRDEKRARALQIPIPVQDIINLPMD
EFNERLSKYDLSETQLSLIRDIRRRGKNKVAAQNCRKRKLDQIVTLADEVKD
MKMRKERLLRDREIIQTERKRIRDKFSALYRHVFQNLRDSEGNPYSQEHWSL
QQSADGTVVLVPRSVDRQQDLTGSNHQQQQQQLGMAGGSGLPHSHHQQQH
LLHLQQHQHPHSQQQHHHGGSSSGSNSTNSSGASGGSQHRPKE
