# Supplementary figures and images for: Molecular Cloning and Expression Profiling of CncC in Bactrocera dorsalis Hendel
Source: Insects. 2022 Aug 30;13(9):785. doi: 10.3390/insects13090785 (PMC9503647; doi:10.3390/insects13090785)

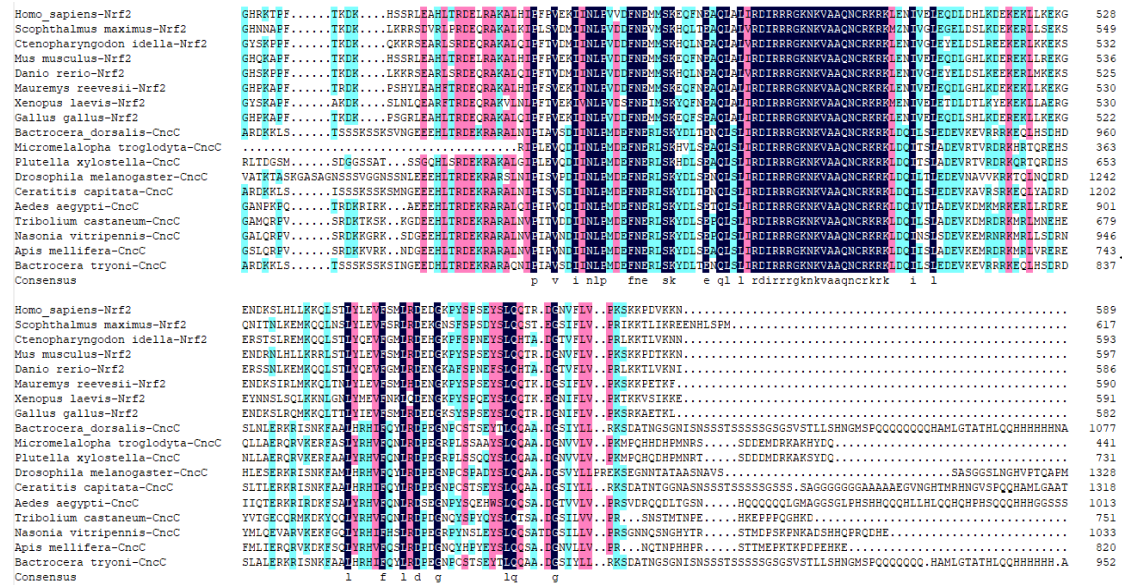

Supplement: Supplementary file 1 [file insects-13-00785-s001.zip › Figure S1 Multiple sequence alignment of conserved regions of insect CncC and Nrf2 from other species.pdf]
