# Supplementary material for: Molecular Cloning and Expression Profiling of CncC in Bactrocera dorsalis Hendel
Source: Insects. 2022 Aug 30;13(9):785. doi: 10.3390/insects13090785 (PMC9503647; doi:10.3390/insects13090785)
Supplement: Supplementary file 1 [file Figure S2 Multiple sequence alignment of conserved regions of CncC of diptera.pdf › Figure S2 Multiple sequence alignment of conserved regions of CncC of diptera.pdf]

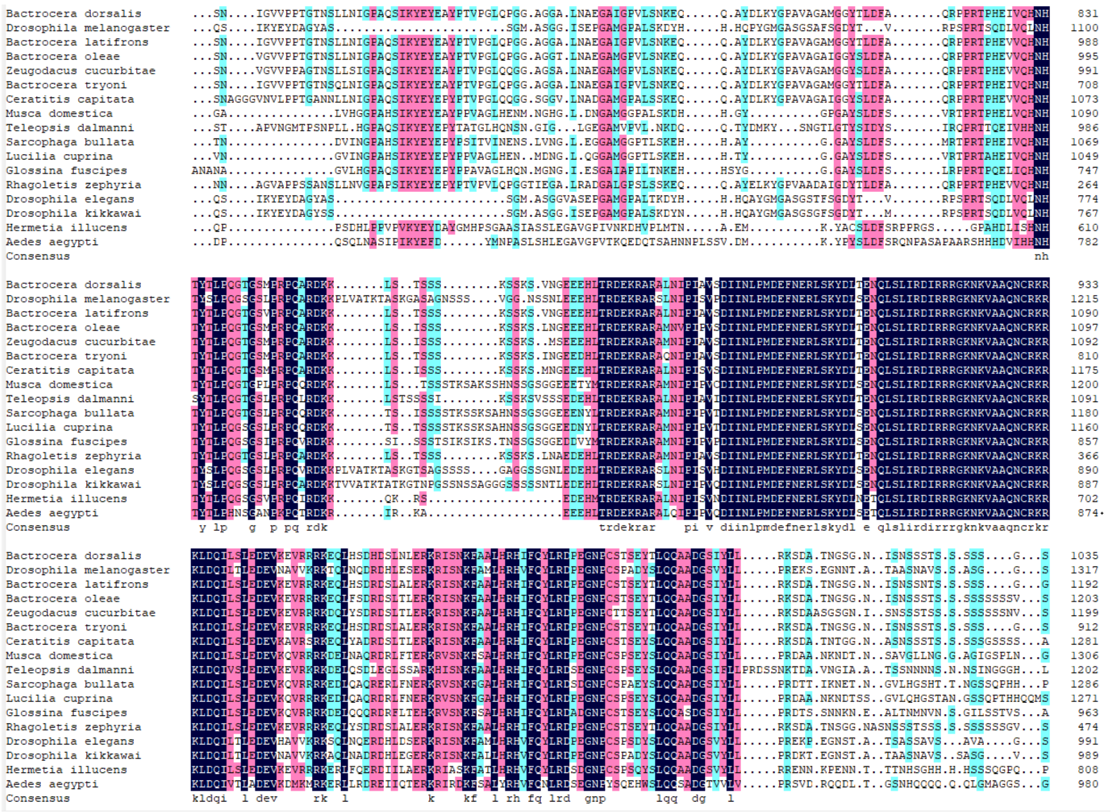

Figure S2. Multiple sequence alignment of conserved regions of *CncC* of diptera. Identical and highly conserved amino acids are indicated by black (100%), pink (75%), and blue (50%), respectively.
